# Renovascular Disease and Its Impact on Endogenous Kidney Repair: Stem/Progenitor Cells

**DOI:** 10.1155/sci/4007512

**Published:** 2026-09-30

**Authors:** Vinaya C. Iyer, Lilach O. Lerman, Alfonso Eirin

**Affiliations:** ^1^ Division of Nephrology and Hypertension Mayo Clinic Rochester Minnesota USA mayo.edu

**Keywords:** progenitor cells, renal repair, renovascular disease, stem cells

## Abstract

**Background:**

Renovascular disease (RVD), a progressive condition characterized by narrowing or occlusion of the renal arteries, is an important cause of secondary hypertension and chronic kidney disease (CKD) in older adults, associated with high cardiovascular morbidity and mortality. Recent evidence suggests that RVD compromises renal repair by impairing complementary vascular, stromal, and tubular repair systems, including endothelial progenitor cells (EPCs), mesenchymal stem/stromal cells (MSCs), and tubular regenerative pathways.

**Summary:**

RVD exposes these endogenous repair cells to multiple injurious stressors, including renal ischemia, hypertension, mechanical stretch, renin–angiotensin–aldosterone system (RASS) activation, and hypoxia. These upstream stressors trigger convergent cellular mechanisms, including oxidative stress, mitochondrial dysfunction, epigenetic and post‐transcriptional (mRNA/miRNA) alterations, endoplasmic reticulum (ER) stress, cellular senescence, and apoptosis, leading to impaired reparative capacity. Cardiovascular comorbidities, including obesity, diabetes, and metabolic syndrome (MetS), further exacerbate these maladaptive responses. Experimental studies suggest that mitoprotective agents, epigenetic and miRNA modulators, ER‐stress inhibitors, and extracellular vesicle (EV)‐based therapies may preserve or restore stem/progenitor cell function.

**Key Messages:**

This review summarizes the current understanding of the impact of RVD on endogenous renal repair, focusing on the complementary roles of EPCs, MSCs, and tubular regenerative pathways, including resident renal progenitor cells, scattered tubular‐like cells (STCs), and injury‐induced epithelial plasticity. We discuss how common pathological stressors converge on shared molecular pathways to impair endogenous repair and highlight emerging therapeutic strategies aimed at preserving or restoring the regenerative capacity of these endogenous repair systems.

## 1. Introduction

Renovascular disease (RVD) is a chronic progressive condition resulting from renal artery stenosis, the narrowing or obstruction of one or both main renal arteries or their major branches. RVD is predominantly due to atherosclerosis, which accounts for 90% of the cases [1]. RVD is present in 6.8% of adults older than 65 years [2], and its prevalence increases with age [3]. Importantly, RVD is strongly associated with cardiovascular disease [4] and incidentally found in patients with peripheral vascular disease [5] and those undergoing routine cardiac catheterization [6].

RVD tends to progress to chronic kidney disease (CKD) and is a major cause of end‐stage kidney disease (ESKD) in the United States [7], accounting for 10% of patients initiating renal replacement therapy with no other identifiable primary cause [8]. In addition, patients with RVD may also develop renovascular hypertension, which accelerates renal injury and increases the risk of potentially lethal complications such as myocardial infarction, flash pulmonary edema, or stroke [9–11].

Current medical management for RVD involves blood pressure control with angiotensin‐converting enzyme inhibitors and angiotensin receptor blockers [12, 13], statins [14], antiplatelet therapy, glycemic control, and lifestyle changes, such as diet, exercise, and smoking cessation, to address cardiovascular risk factors. Although percutaneous transluminal renal angioplasty (PTRA) was a common therapeutic intervention for RVD a few decades ago, large randomized clinical trials, including the Angioplasty and Stenting for Renal Artery Lesions (ASTRAL) [15] and the Cardiovascular Outcomes in Renal Atherosclerotic Lesions (CORAL) [16], failed to demonstrate additional benefits for the prevention of clinical events compared to medical therapy. However, subsequent subgroup analyses and observational studies suggest that selected patients, particularly those with bilateral renal artery stenosis or a solitary functioning kidney resulting in global renal ischemia, rapidly declining renal function, recurrent flash pulmonary edema (Pickering syndrome), or refractory hypertension, may still derive clinically meaningful benefit from renal revascularization when carefully selected [17]. This lack of response to PTRA is largely attributed to the presence of irreversible kidney damage (fibrosis and parenchymal scarring), atheroembolic disease, patient selection, technical factors, and restenosis, among others [17, 18]. These observations underscore the need for additional therapies targeting the mechanisms responsible for renal injury in the post‐stenotic kidney.

The pathophysiology of RVD is complex and involves multiple interactions among injurious pathways within the post‐stenotic kidney. Among them are activation of the renin–angiotensin–aldosterone system (RAAS), inflammation, oxidative stress, and microvascular remodeling, which contribute to hypoxia, fibrosis, and renal dysfunction [19]. In addition, cellular senescence, mitochondrial injury, and apoptosis [20] have also been implicated in the pathogenesis of RVD.

Recent evidence suggests that RVD can compromise renal repair, the kidney’s ability to repair itself after injury or damage, by limiting the potency of stem/progenitor cells (Table 1). RVD exposes endogenous repair cells to multiple pathological stressors, including renal ischemia, hypertension, mechanical stretch, RAAS activation, and hypoxia, which activate convergent molecular mechanisms, including oxidative stress, mitochondrial dysfunction, epigenetic remodeling, post‐transcriptional dysregulation, endoplasmic reticulum (ER) stress, cellular senescence, and apoptosis, that ultimately impair stem/progenitor cell function. RVD also often coexists with other cardiovascular risk factors, such as obesity, diabetes, and metabolic syndrome (MetS), which magnify renal injury and RVD‐induced stem/progenitor cell damage.

**Table 1:** Effects of renovascular disease on endogenous renal repair systems.

Parameter	EPCs	MSCs	Tubular regenerative pathways
Effects of RVD	• Transient enhancement of angiogenic potency during early RVD that deteriorates with disease progression. • Reduced circulating EPC numbers, whereas migratory, proliferative, and tube‐forming capacities remain largely preserved in medically treated patients. • Circulating EPC numbers correlate with stenotic kidney GFR, homing factors, and anti‐inflammatory mediator levels.	• Impairs their ability to repair ischemic murine kidneys in vivo. • Induces epigenetic and post‐transcriptional regulation of mitochondria‐related genes. • Compromises their paracrine function by altering the protein cargo of their EVs (e.g., mitochondrial import, vesicle‐mediated transport, apoptotic, and cell adhesion proteins).	Resident renal progenitor cells/injury‐induced epithelial plasticity: • Identity and relative contribution remain debated. • RVD‐specific effects remain poorly characterized. *Experimentally characterized STCs:* • Increased senescence and SASP markers. • Reduced proliferation and mitochondrial function. • Epigenetic, miRNA, and ER‐stress alterations. • Altered reparative and proangiogenic potency.
Clinical implications	• EPC number and function correlate with disease progression and cardiovascular risk and may serve as biomarkers of vascular health in CKD. • Autologous EPCs have shown renoprotective effects in preclinical RVD models, but clinical studies in RVD remain limited.	• Bone marrow‐, adipose tissue‐, and umbilical cord tissue‐derived MSCs are the principal cell products evaluated clinically. • Early‐phase studies suggest safety and potential renoprotective effects in AKI, CKD, DKD, and RVD. • RVD‐specific clinical trial: autologous adipose‐derived MSCs (NCT01840540; Saad et al., [21]; Abumoawad et al., [22].	• Clinical translation of isolated tubular reparative cells is limited by low abundance, kidney‐tissue procurement, uncertain population identity, and the need for ex vivo expansion. • Injury‐induced epithelial plasticity represents an endogenous process rather than a directly transplantable cell product.
Key references	Chade et al., [23]; Chade et al., [24]; Zhu et al., [25]; Eirin et al., [26]; Chen et al., [27].	Eirin et al., [28]; Saad et al., [21]; Abumoawad et al., [22]; Eirin et al., [19, 29, 30]; Huang et al., [31].	Chen et al., [32]; Nargesi et al., [33, 34]; Farahani et al., [35]; Kazeminia et al., [36]; Kazeminia et al., [37, 38].

Abbreviations: EPCs, endothelial progenitor cells; ER, endoplasmic reticulum; EVs, extracellular vesicles; GFR, glomerular filtration rate; MSCs, mesenchymal stem/stromal cells; SASP, senescence‐associated secretory phenotype; STCs, scattered tubular‐like cells.

This review aims to synthesize current knowledge on the impact of RVD on endogenous renal repair, focusing on endothelial progenitor cells (EPCs), mesenchymal stem/stromal cells (MSCs), and tubular regenerative pathways, including resident renal progenitor cells, scattered tubular‐like cells (STCs), and injury‐induced epithelial plasticity (Figure 1), and to discuss potential mechanisms of RVD‐induced repair system dysfunction and novel strategies to preserve or enhance reparative capacity. We searched the PubMed database for English‐language articles published between 2000 and 2026 using the terms “endothelial progenitor cells,” “mesenchymal stem/stromal cells,” “scattered tubular‐like cells,” “renal progenitor cells,” “tubular regeneration,” “epithelial dedifferentiation,” “CD24,” “CD133,” and “Pax2.” Much of the current evidence derives from a limited number of research groups and preclinical RVD models, highlighting the need for independent validation and clinical translation. We focused on three complementary, but biologically distinct, arms of endogenous renal repair: vascular repair mediated by EPCs, stromal, and paracrine support mediated by MSCs/pericytes, and tubular regenerative pathways. The latter encompass resident renal progenitor cells, STCs, and transient injury‐induced epithelial plasticity, which represent related but not necessarily biologically equivalent concepts. Together, these vascular, interstitial, and tubular repair systems provide an integrated framework for understanding how RVD disrupts coordinated kidney regeneration and identify potential therapeutic targets for preserving endogenous renal repair.

**Figure 1:**
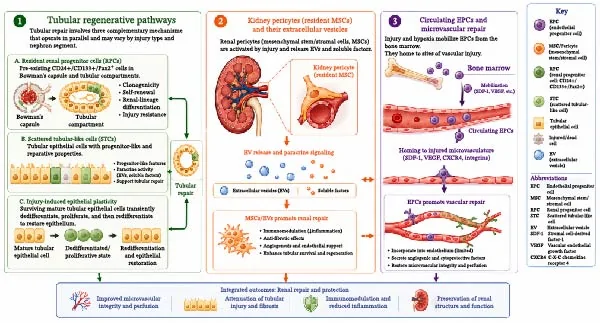
Endogenous renal repair: complementary vascular, perivascular, and tubular regeneration pathways. These endogenous cell populations sense kidney injury, and when activated, home to sites of injury where they promote renal repair through paracrine and regenerative mechanisms. Endogenous renal repair is mediated by complementary vascular, perivascular, and tubular progenitor‐like cell populations: Renal injury activates three complementary endogenous repair systems that promote tissue regeneration through distinct but interconnected mechanisms. (1) Tubular regenerative pathways: current evidence supports three complementary, but biologically distinct, mechanisms contributing to tubular repair. Resident renal progenitor cells (RPCs), characterized by expression of CD24, CD133, and Pax2, reside in Bowman’s capsule and tubular compartments and exhibit clonogenicity, self‐renewal, and renal‐lineage differentiation potential. Scattered tubular‐like cells (STCs) represent a tubular epithelial population with progenitor‐like and reparative properties that contributes predominantly through paracrine signaling and extracellular vesicle (EV) secretion. In parallel, surviving mature tubular epithelial cells can undergo transient dedifferentiation after injury, activating a regenerative transcriptional program before redifferentiating into functional tubular epithelium. The relative contribution and lineage relationship among these three mechanisms remain areas of active investigation. Together, these complementary mechanisms support tubular regeneration while preserving tubular integrity and limiting fibrosis. (2) Kidney pericytes (resident mesenchymal stem/stromal cells, MSCs): Pericytes residing within the perivascular niche become activated after injury and release EVs, cytokines, and growth factors that exert immunomodulatory, anti‐inflammatory, anti‐fibrotic, and pro‐angiogenic effects, thereby preserving the renal microvasculature and promoting tissue repair. (3) Circulating endothelial progenitor cells (EPCs): Ischemia and hypoxia stimulate mobilization of EPCs from the bone marrow through homing signals such as stromal cell‐derived factor‐1 (SDF‐1) and vascular endothelial growth factor (VEGF). EPCs home to injured renal microvessels, where they promote endothelial repair primarily through paracrine signaling and secretion of angiogenic and vasculoprotective mediators, with limited direct incorporation into injured endothelium. Collectively, these complementary cellular and cell‐free mechanisms improve renal perfusion, preserve microvascular integrity, attenuate tubular injury and fibrosis, reduce inflammation, and maintain renal structure and function.

## 2. Main Text

### 2.1. EPCs

EPCs were first described in 1997 as circulating bone marrow‐derived mononuclear cells that are mobilized in response to ischemia and contribute to endothelial repair and neovascularization in ischemic tissues [39, 40]. These cells are characterized by the expression of both hematopoietic and endothelial markers, including CD34, kinase insert domain receptor (KDR), CD146, VE cadherin, and von Willebrand factor. EPCs can be classified according to their appearance in culture into two principal subpopulations: “early EPCs,” now referred to as “circulating angiogenic cells (CACs)” and “late‐outgrowth EPCs,” currently known as “endothelial colony‐forming cells (ECFCs).” CACs are observed after 4–7 days in culture and express immature hemopoietic stem cell markers, such as CD133, whereas ECFCs appear in culture after 14–21 days as cobblestone‐shaped colonies with high rates of proliferation [41]. However, the relationship between CACs and ECFCs remains controversial because they differ in origin, phenotype, and mechanism of action [42, 43]. Both CACs and ECFCs have clinical relevance as they promote neovascularization in ischemic conditions, and changes in their levels and functions may serve as diagnostic or prognostic markers for cardiovascular disease [42].

Previous studies in mice subjected to unilateral renal artery clamping have shown that EPCs are mobilized and home to ischemic kidneys by day 7 after ischemia [44]. Mechanical stretch is an important stimulus for EPC mobilization and involves bone marrow stromal cell‐derived factor (SDF)‐1 synthesis and matrix metalloproteinase (MMP)‐9 activation in a p47phox‐dependent manner [45]. In line with this, previous studies in swine RVD have shown that mobilization of EPCs from the bone marrow is regulated by the release of several growth factors from the post‐stenotic kidney, including the homing factors SDF‐1 and stem cell factor, which bind cognate receptors expressed by EPCs [23]. These findings were subsequently confirmed in patients with RVD, in whom the release of inflammatory mediators and homing factors was accompanied by a negative EPC gradient across the stenotic kidney, suggesting EPC retention within the injured kidney to promote renal repair. However, release of homing factors (e.g., SDF‐1) is not specific of RVD as they have also been reported in response to ischemia/reperfusion acute kidney injury (AKI) [46].

The potential of EPCs to repair damaged kidneys is underscored by several experimental studies showing that the exogenous delivery of EPCs ameliorates renal injury and functional deterioration. For example, a single intrarenal infusion of autologous ECFCs preserved microvascular architecture and function and decreased microvascular remodeling in swine RVD [23, 24]. Importantly, EPCs increased the expression of potent angiogenic factors in the post‐stenotic kidney. Among them is vascular endothelial growth factor (VEGF), which stimulates the proliferation and maturation of new vessels. Indeed, intrarenal administration of VEGF has been shown to protect the post‐stenotic swine kidney [47], highlighting the role of this cytokine to preserve the renal microcirculation and, consequently, decreasing progressive renal injury in chronic RVD. In addition, EPCs increased the renal expression of phosphorylated endothelial nitric oxide synthase (eNOS), which promotes microvascular sprouting by maintaining vasodilation during the early steps of angiogenesis [48]. Delivery of autologous EPCs into the pig stenotic kidney was associated with increased numbers of Oct‐4‐positive tubular cells, suggesting activation of regenerative pathways [49], and decreased expression of transforming growth factor (TGF)‐β and MMP‐2, suggesting decreased fibrogenic activity and improved matrix turnover. Furthermore, cultured ECFCs released extracellular vesicles (EVs) in vitro and induced a VEGF‐dependent phenotypic switch (M1‐to‐M2) in cultured monocytes [26]. These observations suggest additional anti‐inflammatory properties via horizontal transfer of messenger RNA (mRNA) from EPCs to inflammatory cells.

EPCs have also demonstrated important renoprotective effects when administered in conjunction with renal revascularization. Combining PTRA with intra‐renal delivery of autologous ECFCs improved swine stenotic‐kidney hemodynamics, function, and microvascular remodeling and decreased serum creatinine levels more effectively than PTRA alone [26]. Furthermore, addition of EPCs restored medullary oxygen‐dependent tubular function and attenuated oxidative stress, inflammation, and tubulointerstitial fibrosis, underscoring their ability to preserve the structure and function of revascularized kidneys.

However, previous studies suggest that RVD can induce detrimental effects on EPCs. Swine EPCs may undergo functional changes during exposure to RVD [25]. Specifically, EPC function is transiently enhanced during the early stage of experimental RVD (3 weeks), characterized by increased proliferative and tube‐forming capacities and a proangiogenic profile of circulating growth factors, including VEGF, and homing signals for EPC migration to injury sites, such as SDF‐1, which contribute to compensatory vascular adaptation and repair. This early adaptive response of RVD is likely secondary to a transient activation of the RAAS. Angiotensin II infusion in mice increases local and systemic expression of VEGF and its receptors [50] and potentiates VEGF‐induced human EPC proliferation and tube formation [51]. Furthermore, angiotensin II may also directly promote VEGF‐induced eNOS activation [52], underscoring the role of this peptide hormone in stimulating the EPC angiogenic potential in the early stage of RVD. However, this adaptive angiogenic response progressively declines with disease progression and returns to baseline after ~12 weeks of experimental RVD. This was accompanied by decreased levels of angiotensin II, which was replaced by other pressor mechanisms, and downregulation of eNOS likely mediated by increased oxidative stress.

In subsequent studies, ECFCs were isolated from the inferior vena cava and renal veins of medically treated patients with RVD. The number of ECFCs was reduced in the systemic circulation compared with normotensive controls and was further reduced in the stenotic kidney. Despite this reduction, their migratory, proliferative, and tube‐forming capacities were preserved, possibly because these patients were all treated with RAAS inhibitors [27]. Similarly, most RVD patients were treated with statins and calcium channel blockers, which might also increase EPC mobilization and differentiation and attenuate EPC apoptosis [53]. Importantly, the numbers of renal vein EPCs correlated directly with the stenotic kidney glomerular filtration rate (GFR), EPC homing factors, and anti‐inflammatory mediator levels, implying that the milieu of RVD exerts potentially meaningful effects on the function of EPCs recruited to the stenotic kidneys. These observations suggest that EPC reparative capacity remains largely preserved during the early stages of RVD and in medically treated patients, whereas prolonged ischemia, persistent oxidative stress, and disease progression may ultimately compromise the EPC function. Accordingly, strategies aimed at preserving the EPC number and function may be most effective before irreversible vascular injury develops. These findings support the concept that EPC biology evolves dynamically during RVD progression, with an early compensatory phase characterized by preserved or enhanced angiogenic function, followed by progressive functional decline as ischemic injury becomes chronic.

### 2.2. MSCs

Having discussed the impact of RVD on EPCs, we now turn to MSCs. MSCs are multipotent adult nonembryonic stem cells that exhibit a fibroblast‐like, spindle‐shaped morphology and fulfill the minimal criteria established by the International Society for Cellular Therapy, including expression of CD44, CD73, CD90, and CD105; lack of expression of hematopoietic markers (e.g., CD45, CD34, CD14, and CD11b); trilineage differentiation potential (adipocytes, chondrocytes, and osteocytes); and plastic adherence under standard culture conditions [54].

MSCs reside in the perivascular niche of many organs, and their renal counterparts are thought to include pericytes, which act as a reservoir of undifferentiated cells to address the repair needs of the kidney [55]. Notably, MSCs are endowed with important anti‐inflammatory, immunomodulatory, and pro‐angiogenic features, which contribute to improving functional and structural recovery of renal tubular, glomerular, and interstitial compartments exposed to AKI and CKD [56]. For example, Kramann et al. found that selective ablation of Gli1^+^ kidney pericytes caused loss of peritubular capillaries and proximal tubular injury, providing direct evidence that pericytes are required to maintain renal microvascular integrity and protect tubular epithelial cells [57]. Similarly, studies from Joosten’s group demonstrated that umbilical cord‐derived MSCs promote a memory‐like CD4^+^ T‐cell phenotype while maintaining their immunomodulatory properties [58], whereas van Koppen et al. [59] showed that conditioned medium derived from human embryonic MSCs rescued kidney function in rats with established CKD. Systemic delivery of porcine kidney‐derived pericytes improved renal perfusion, GFR, and medullary oxygenation in stenotic kidneys of mice subjected to unilateral RVD [60]. Similarly, in swine RVD, intrarenal delivery of allogeneic adipose‐tissue‐derived MSCs in combination with renal revascularization preserved the structure and function of the ischemic kidney. Delivery of autologous adipose‐tissue‐derived MSCs without renal revascularization exerted comparable effects in the stenotic kidney. Importantly, these renoprotective effects were subsequently translated to patients with RVD. Autologous adipose‐derived MSCs infused through the renal artery, in the presence or absence of renal revascularization, improved renal perfusion and blood flow, and attenuated hypoxia and inflammatory injury beyond the stenotic lesion [21], although improvements were observed earlier in preclinical models (4 weeks) than in clinical studies (3 months after MSC administration).

MSCs exert their protective effects largely through paracrine function, including the secretion of cytokines, growth factors, and EVs enriched with mRNAs, miRNAs, and proteins capable of modulating several pathways responsible for renal injury. Swine adipose tissue‐derived MSCs actively secrete VEGF in the cell culture. A single intra‐renal delivery of EVs derived from autologous adipose‐tissue‐derived MSCs improved medullary oxygenation and fibrosis and restored renal blood flow and GFR in pigs with renal artery stenosis [61]. miRNA‐ and mRNA‐sequencing analyses of their cargo confirm that they transport gene regulatory information to modulate angiogenesis, adipogenesis, and other cell pathways in recipient cells [62]. These include vasculoprotective genes, which preserve the stenotic kidney microvasculature, as well as components of tumor necrosis factor‐α‐induced gene/protein (TSG)‐6 and TGF‐β [63] signaling, which regulate macrophage function and phenotype. Importantly, the potential of autologous MSC‐derived EVs to decrease renal inflammation is partly attributed to their cargo of interleukin (IL)‐10 as delivery of IL‐10‐silenced EVs failed to preserve the structure and function of swine RVD kidneys [61].

However, RVD also compromises the biological properties and reparative potential of MSCs. RVD impairs the capacity of human adipose tissue‐derived MSCs to repair murine post‐stenotic kidneys partly by post‐transcriptional regulation of mitochondria‐related genes [29]. Specifically, MSCs harvested from healthy volunteers improved post‐stenotic kidney GFR and attenuated vascular loss, tubular injury, and fibrosis. However, these salutary effects were abrogated in mice treated with MSCs obtained from patients with RVD. Importantly, inhibition of miR‐378h, which primarily targets mitochondria‐related genes, restored the expression of mitochondria‐related target genes, attenuated mitochondrial impairment of human RVD‐MSCs in vitro, and restored their ability to repair murine post‐stenotic kidneys in vivo.

RVD also impairs the paracrine function of MSCs by altering the molecular cargo of their EVs. Proteomic analysis showed that although healthy volunteer‐ and RVD‐MSCs released similar numbers and sizes of EVs, several proteins were dysregulated in RVD‐MSCs [29]. This included dysregulation of proteins involved in mitochondrial import (e.g., TOMM7), vesicle‐mediated transport, apoptosis, and cell adhesion. Similarly, in vitro *s*tudies have shown that MSCs obtained from patients with RVD exhibit greater levels of senescence‐associated DNA damage and reduced migration capacity compared to those harvested from healthy individuals, associated with lower expression of angiogenic proteins such as VEGF and hepatocyte growth factor [64]. Collectively, these studies indicate that RVD induces both intrinsic mitochondrial dysfunction and defective paracrine signaling in MSCs, supporting the development of therapeutic strategies aimed at preserving or restoring MSC reparative potency.

### 2.3. Tubular Regenerative Pathways

Tubular repair involves several related but biologically distinct regenerative mechanisms. For conceptual clarity, this section distinguishes: (1) resident renal progenitor cells, including CD24^+^/CD133^+^/Pax2^+^ populations identified in the adult human kidney; (2) STCs, as defined in the STC literature; and (3) transient dedifferentiation and regenerative reprograming of surviving tubular epithelial cells following injury. These mechanisms may operate in parallel, but current evidence does not establish that they represent the same cell population or biological process.

RVD may impair multiple components of tubular regenerative pathways. Following AKI, surviving differentiated tubular epithelial cells can undergo transient dedifferentiation and activate regenerative transcriptional programs that support epithelial repair [65–69]. This injury‐induced epithelial plasticity should be distinguished from resident CD24^+^/CD133^+^ renal progenitor populations and from STCs as defined in the human tubular cell literature. The molecular pathways regulating these processes include cell‐cycle re‐entry, Wnt signaling, hypoxia‐responsive pathways, inflammatory cytokines, and activation of sex‐determining region Y‐box transcription factors, although their specific relevance to RVD remains incompletely defined.

#### 2.3.1. Resident Renal Progenitor Cells

Resident CD24^+^/CD133^+^ renal progenitor cells have been identified in Bowman’s capsule and tubular compartments of the adult human kidney [70]. These cells exhibit clonogenicity, self‐renewal, resistance to injury, and renal‐lineage differentiation potential and possess transcriptional and miRNA profiles distinct from mature proximal tubular epithelial cells. Pax2^+^ tubular progenitor populations pre‐existing before injury have also been shown to exhibit enhanced stress resistance and clonal expansion during repair.

#### 2.3.2. STCs

STCs, as described by Smeets, Hansson, and colleagues, are scattered tubular epithelial cells with distinctive morphological, molecular, and functional characteristics [71–73]. They are smaller and contain fewer mitochondria than terminally differentiated tubular epithelial cells and express the glycosyl phosphatidylinositol‐anchored protein CD24 and the pentaspanning transmembrane glycoprotein CD133, as well as vimentin, COL7A1, and claudin‐1. Because CD24 and CD133 are not specific to STCs, these markers alone do not establish the cell identity.

#### 2.3.3. Injury‐Induced Epithelial Plasticity

Mouse lineage‐tracing studies demonstrate that surviving differentiated tubular epithelial cells can re‐enter the cell cycle and regenerate damaged nephron segments through self‐duplication [74, 75]. Following injury, these cells may transiently express markers associated with regenerative or dedifferentiated states. However, these studies did not directly establish that transiently dedifferentiated epithelial cells are identical to the resident human CD24^+^/CD133^+^/Pax2^+^ progenitors or to STCs defined by morphological and functional criteria.

Taken together, current evidence supports three complementary but biologically distinct components of tubular regenerative pathways. First, resident CD24^+^/CD133^+^ (Pax2^+^) renal progenitor cells have been identified in the adult human kidney, where they exhibit clonogenicity, self‐renewal, resistance to injury, and renal‐lineage differentiation potential [70, 76–78]. Second, STCs, as originally described by Smeets et al. [65] and Hansson et al. [71], represent a tubular epithelial population with progenitor‐like morphological and functional characteristics. Third, lineage‐tracing studies in mice demonstrate that surviving differentiated tubular epithelial cells can transiently activate a regenerative transcriptional program after injury and express markers commonly associated with STCs, including CD24 and CD133 [74, 75]. The relative contribution of these tubular regenerative pathways is likely to vary according to the species, nephron segment, injury type, and disease stage. Their precise lineage relationships remain unresolved, and they should not be treated as interchangeable populations.

Despite unresolved lineage relationships, experimental studies demonstrate that isolated renal progenitor cells and STCs possess reparative properties in models of kidney injury. In murine models of AKI, systemic administration of STCs decreased morphologic kidney damage and improved renal function. For example, Bussolati et al. [79] found that intravenous injection of adult human renal progenitor cells in severe combined immunodeficiency (SCID) mice with glycerol‐induced tubulonecrosis homed into the injured kidney and integrated in tubules. Similarly, Sagrinati et al. [70] found that injection of CD24^+^/CD133^+^ renal progenitor cells into SCID mice that had AKI resulted in the regeneration of tubular structures of different portions of the nephron. Studies from Romagnani’s group showed that tubular lineage‐committed renal progenitor cells engrafted injured mouse kidneys and contributed to tubular repair [77]. Administration of CD133^+^ renal progenitor cells promoted restoration of the renal tissue while reducing markers of tubular injury and inflammation. In line with this, delivery of swine STCs into the aorta of 2 kidneys, 1‐clip (2k,1c) mice attenuated renal hypoxia, fibrosis, tubular injury, and capillary loss and improved renal perfusion, blood flow, and GFR [32], underscoring the capacity of these cells to repair damaged kidneys.

The mechanisms of STC‐induced renoprotection are multifactorial and are likely to involve renal engraftment, endothelial differentiation, and paracrine effects. Cultured STCs secrete several proangiogenic cytokines, including VEGF, at levels comparable to MSCs [80], upregulate the expression of vascular development genes [81], and increase renal expression of VEGF and intrarenal vessel density in mice with AKI [70, 82] and CKD [32]. In addition, studies from Camussi’s group found that STCs synthesize and release erythropoietin under hypoxia [83], whereas Sallustio et al. [84] found that they secrete factors involved in the cell regeneration process, such as inhibin‐A and decorin to protect physically injured or chemically damaged renal proximal tubular epithelial cells. Furthermore, delivery of STC‐derived EVs improves renal perfusion and oxygenation and ameliorates inflammatory and profibrotic gene expression in ischemic murine kidneys.

Like EPCs and MSCs, STCs are susceptible to the deleterious effects of RVD, which can compromise their integrity and their potential to repair damaged kidneys. STCs isolated from swine RVD kidneys exhibit increased expression of senescence genes and markers of senescence‐associated secretory phenotype (SASP), a cocktail of inflammatory factors released from senescent cells [85]. This was accompanied by lower capacity to ameliorate murine ischemic injury compared to STCs harvested from normal pigs [32]. Swine RVD‐STCs have impaired proliferative potential and mitochondrial structural and functional abnormalities [33], likely due to downregulation of genes implicated in the cell cycle and post‐transcriptional silencing of mitochondria‐related genes. Importantly, these molecular and structural alterations impaired the ability of STCs to improve the viability of injured renal tubular epithelial cells in vitro, underscoring their vulnerability in the setting of RVD.

RVD‐induced epigenetic alterations may further contribute to limiting the reparative potential of STCs. RVD induces epigenetic changes in apoptosis‐, proteolysis‐, and mitochondria‐related genes, which correlate with alterations in the transcriptomic profile and the corresponding function of swine STCs. Likewise, RVD induces site‐specific changes in the methylation and hydroxymethylation profile of inflammatory genes in STCs, which are linked to transcriptional activation and the production of inflammatory cytokines [36]. Finally, RVD alters the expression of vasculature‐ and ER stress‐related genes in swine STCs, impairing their proangiogenic activity [37] and reparative potency [38]. Collectively, these findings indicate that RVD progressively impairs the reparative capacity of STCs through convergent mechanisms involving mitochondrial dysfunction, cellular senescence, epigenetic remodeling, and ER stress, thereby limiting endogenous tubular repair.

### 2.4. Mechanisms of RVD‐Induced Stem/Progenitor Cell Dysfunction: A Convergent Pathogenic Framework

Although EPCs, MSCs, and the cellular components of tubular regenerative pathways occupy distinct anatomical niches and perform different reparative functions, accumulating evidence suggests that they are affected by overlapping cellular stress responses in RVD (Figure 2). RVD exposes endogenous repair cells to a hostile microenvironment characterized by chronic ischemia, reduced oxygen delivery, hypertension‐induced mechanical stretch, persistent activation of the RAAS, oxidative stress, inflammation, and, frequently, superimposed cardiovascular risk factors such as obesity, diabetes mellitus, and MetS [19]. Rather than acting independently, these insults converge on a limited number of intracellular pathways that ultimately impair stem/progenitor cell viability and regenerative capacity. These convergent pathways can be grouped into four interconnected biological axes: (1) hypoxia–mitochondrial dysfunction–reactive oxygen species (ROS), (2) RAAS activation/mechanical stretch–oxidative stress–endothelial dysfunction, (3) epigenetic and post‐transcriptional reprograming, and (4) ER stress, cellular senescence, and impaired paracrine signaling.

**Figure 2:**
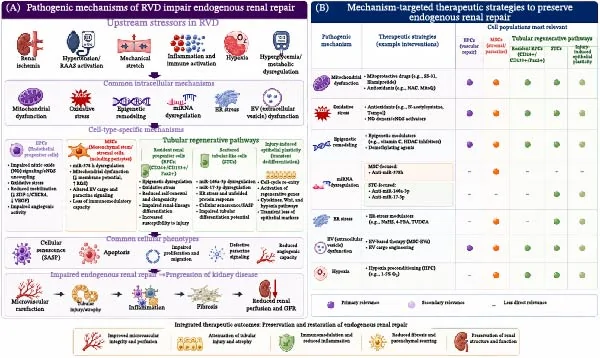
Mechanisms of renovascular disease‐induced endogenous repair cell dysfunction and therapeutic strategies to preserve renal repair. (A) Convergent mechanisms of endogenous repair cell dysfunction in renovascular disease (RVD). RVD exposes endogenous repair systems to multiple injurious stressors, including renal ischemia, hypertension‐induced mechanical stretch, activation of the renin–angiotensin–aldosterone system (RAAS), renal hypoxia, and cardiovascular risk factors such as obesity, diabetes mellitus, and metabolic syndrome (MetS). These upstream stressors converge on common intracellular mechanisms, including mitochondrial dysfunction, oxidative stress, miRNA dysregulation, epigenetic remodeling, endoplasmic reticulum (ER) stress, and alterations in extracellular vesicle (EV) cargo. Although many of these mechanisms are shared, they affect endogenous repair systems in a cell‐specific manner. EPC dysfunction is characterized predominantly by impaired nitric oxide signaling, oxidative stress, defective mobilization, and reduced angiogenic activity. MSC dysfunction is closely associated with mitochondrial injury, miR‐378h dysregulation, altered EV cargo, impaired paracrine signaling, and reduced immunomodulatory capacity. Within tubular regenerative pathways, current evidence derives predominantly from studies of resident renal progenitor cells (RPCs) and experimentally characterized STCs, whereas the molecular mechanisms governing injury‐induced epithelial plasticity remain less well defined. ER stress, cellular senescence, and dysregulation of miR‐146a‐3p and miR‐17‐3p have been most extensively characterized in STCs. Activation of these pathways promotes cellular senescence, apoptosis, impaired proliferation and migration, defective paracrine signaling, and reduced regenerative capacity, ultimately compromising endogenous renal repair and leading to microvascular rarefaction, tubular injury, inflammation, fibrosis, reduced renal perfusion and glomerular filtration rate (GFR), and progression of chronic kidney disease. (B) Mechanism‐targeted therapeutic strategies to preserve endogenous renal repair. Therapeutic interventions target both common pathogenic pathways shared among EPCs, MSCs, and tubular regenerative pathways, as well as mechanisms that predominate in specific repair cell populations. Mitoprotective agents preserve mitochondrial integrity and cellular bioenergetics; epigenetic modifiers and miRNA‐targeted therapies restore gene regulatory networks; ER stress modulators improve proteostasis; and EV‐based therapies restore reparative paracrine signaling. Cell‐specific strategies include inhibition of miR‐378 h to improve MSC mitochondrial function and modulation of miR‐146a‐3p and miR‐17‐3p to enhance the proangiogenic phenotype of experimentally characterized STCs. Additional approaches, including hypoxia preconditioning, induced pluripotent stem cell (iPSC)‐derived renal and vascular cells, and kidney organoids, represent emerging regenerative platforms that may overcome limitations associated with autologous cell therapy. By preserving or restoring endogenous repair mechanisms across the vascular, perivascular, and tubular compartments of the kidney, these interventions have the potential to improve microvascular integrity, enhance tubular regeneration, attenuate inflammation and fibrosis, and ultimately preserve renal structure and function.

#### 2.4.1. Upstream Stressors

Renal ischemia is the initiating event in RVD and triggers tissue hypoxia, nutrient deprivation, and adenosine triphosphate (ATP) depletion. Chronic hypoxia compromises mitochondrial oxidative phosphorylation, decreases electron transport chain activity, and increases the production of ROS, thereby initiating oxidative damage to proteins, lipids, and mitochondrial DNA [86]. Hypertension further exacerbates this process through cyclic mechanical stretch, which activates NADPH oxidases and amplifies ROS generation [87]. Persistent RAAS activation, particularly angiotensin II signaling, further promotes oxidative stress by stimulating NADPH oxidase activity, impairing endothelial nitric oxide bioavailability, inducing mitochondrial dysfunction, and activating inflammatory and apoptotic pathways [88, 89]. These pathological processes are further amplified in patients with obesity, diabetes, or MetS, in whom hyperglycemia, insulin resistance, dyslipidemia, and lipotoxicity create an additional metabolic burden that accelerates cellular dysfunction.

#### 2.4.2. Mitochondrial Dysfunction as a Central Hub

Among the downstream consequences of these insults, mitochondrial dysfunction appears to represent a central pathogenic hub that integrates multiple upstream stressors into stem/progenitor cell failure. Mitochondria regulate ATP production, redox homeostasis, calcium signaling, apoptosis, and cellular metabolism, all of which are essential for stem cell maintenance and tissue repair [90, 91]. Experimental studies demonstrate mitochondrial structural abnormalities, impaired membrane potential, decreased ATP generation, excessive ROS production, and altered mitochondrial dynamics in both MSCs and STCs exposed to RVD or MetS [33]. In addition, post‐transcriptional regulation of mitochondria‐related genes by miRNAs, including miR‐378h in MSCs, and epigenetic modifications affecting mitochondrial pathways further compromise mitochondrial integrity and cellular bioenergetics [29]. Although direct evidence in EPCs remains comparatively limited, oxidative stress and reduced eNOS bioavailability similarly impair EPC survival, proliferation, and angiogenic capacity [25, 27], suggesting that mitochondrial injury may represent a shared mechanism across vascular, stromal, and tubular repair systems.

#### 2.4.3. Epigenetic and Post‐Transcriptional Regulation

Increasing evidence indicates that RVD not only induces acute cellular stress but also reprograms stem/progenitor cells through persistent epigenetic and post‐transcriptional mechanisms. DNA methylation, hydroxymethylation, histone modifications, and dysregulated miRNA expression alter transcriptional programs controlling mitochondrial function, angiogenesis, inflammation, apoptosis, cell‐cycle progression, and senescence. These changes have been extensively characterized in MSCs and STCs, where RVD and MetS induce widespread epigenetic remodeling associated with impaired reparative potency [30, 36]. In parallel, alterations in EV‐associated miRNAs and mRNAs modify intercellular communication, diminishing the ability of endogenous repair cells to transfer pro‐regenerative signals to injured renal tissue [30]. Collectively, these findings suggest that epigenetic and miRNA dysregulation provide a mechanistic link between chronic ischemic injury and the long‐lasting impairment of endogenous renal repair.

#### 2.4.4. ER Stress, Proteostasis, and Cellular Senescence

Another convergent mechanism is the disruption of ER homeostasis. Increased oxidative stress, ATP depletion, and accumulation of misfolded proteins activate the unfolded protein response, which initially promotes adaptation but ultimately induces apoptosis when ER stress becomes persistent [92]. Recent studies demonstrate that RVD activates ER stress pathways in STCs, reducing their angiogenic and reparative potential [38]. ER stress has also been implicated in MSC dysfunction under metabolic conditions [93]. In parallel, sustained oxidative and mitochondrial stress promote cellular senescence, characterized by irreversible cell‐cycle arrest and acquisition of the SASP [85, 94]. Senescent EPCs exhibit impaired proliferation and angiogenesis [95, 96], senescent MSCs display reduced migration and paracrine activity [64], and senescent STCs lose their capacity to regenerate injured tubular epithelium while releasing pro‐inflammatory cytokines that further amplify renal injury [32]. Therefore, senescence represents a common downstream phenotype through which chronic RVD progressively exhausts endogenous repair mechanisms.

#### 2.4.5. Integrated Downstream Phenotype

Collectively, these converging mechanisms impair EPC‐mediated vascular repair, MSC‐mediated stromal/paracrine support, and tubular regenerative pathways. EPCs exhibit impaired mobilization, migration, proliferation, and angiogenesis, limiting the preservation of the renal microvasculature [23, 25, 26]. MSCs develop mitochondrial dysfunction, altered immunomodulatory activity, defective EV cargo, and diminished paracrine signaling, reducing their ability to suppress inflammation and promote vascular repair [29, 31, 61]. Within the tubular compartment, RVD‐exposed STCs undergo mitochondrial dysfunction, ER stress, epigenetic reprograming, and senescence, impairing tubular regeneration and enhancing inflammatory signaling [32, 36, 38]. Thus, these convergent pathways drive a progressive transition from an adaptive regenerative phenotype to a dysfunctional cellular state, ultimately compromising endogenous kidney repair and accelerating CKD progression.

Taken together, current evidence supports a unified model in which renal ischemia, hypertension, hypoxia, RAAS activation, oxidative stress, and metabolic abnormalities converge on mitochondrial dysfunction, epigenetic remodeling, miRNA dysregulation, ER stress, and EV dysfunction, which collectively drive apoptosis, senescence, impaired paracrine signaling, and loss of regenerative capacity across vascular, stromal, and tubular repair systems [19, 55]. This integrated framework identifies shared therapeutic targets, including mitochondrial protection, epigenetic modulation, ER stress inhibition, restoration of EV cargo, and miRNA modulation, that may preserve endogenous renal repair despite differences in the stem/progenitor cell lineage.

### 2.5. Cardiovascular Risk Factors

Patients with RVD often present with cardiovascular risk factors, including obesity, diabetes, and MetS, which are associated with poor renal outcomes and may also compromise endogenous repair systems. Obesity is an epidemic condition associated with the initiation and progression of preexisting CKD [97] and an independent risk factor for ESKD [98]. Substantial evidence demonstrates that obesity not only contributes to atherosclerosis that initiates RVD but also aggravates kidney damage directly, thereby worsening both structural and functional outcomes. Diabetes is a major cause of ESKD [99], accounting for one‐third of incident cases worldwide [100]. The prevalence of RVD is greater in diabetic patients compared to the general population [101, 102] and is associated with several therapeutic challenges [103]. Similarly, MetS, which encompasses a group of cardiovascular risk factors, including hyperlipidemia, central obesity, insulin resistance, and hypertension [104], frequently coexists with RVD [105]. Notably, the coexistence of MetS and RVD is associated with more severe post‐stenotic injury, including mitochondrial damage, medullary hypoxia, microvascular loss, and fibrosis [34, 106].

Obesity, diabetes, and MetS could also compromise endogenous repair mechanisms, aggravating post‐ischemic kidney injury. For example, studies in mice have shown that high‐fat diet‐induced obesity suppresses the circulating levels of EPCs and impairs their angiogenic and migratory function [107]. EPC colony‐forming capacity is significantly lower in individuals with low high‐density lipoprotein levels, likely through modulation of EPC apoptosis via caspase‐3 activity [108]. The adhesive, migratory, and angiogenic capacity of ECFCs are also impaired in obese individuals [95], further supporting the detrimental impact of obesity on the functional properties of EPCs. EPC numbers are also reduced in children with type‐1 diabetes [109], whereas their proliferative and tube‐forming capabilities are impaired in adults with type‐2 diabetes and inversely correlate with glycated hemoglobin A1c levels [96], suggesting that poor glycemic control may contribute to EPC dysfunction.

Cardiovascular risk factors also compromise the integrity and function of MSCs. High glucose levels induce MSC senescence by altering the expression of phosphorylated (p‐)phosphatidylinositol 3‐kinase/protein kinase B and p‐mammalian target of rapamycin signaling [93]. Adipose tissue‐derived MSCs harvested from obese pigs display enhanced adipogenic and osteogenic abilities and increased senescence. Interestingly, despite these metabolic alterations, their immunomodulatory potential remained largely preserved. In agreement, studies in patients with diabetic kidney disease have shown that the functionality, trophic factor secretion, as well as the angiogenic and immunomodulatory activities of adipose tissue‐derived MSCs remained intact despite alterations in the transcriptome and migratory function. Collectively, these findings suggest that although diabetes alters MSC transcriptomic and migratory profiles, several key reparative functions remain relatively preserved.

MetS induces mitochondrial structural abnormalities and dysfunction in swine MSCs, likely mediated by miRNA‐ and epigenetic‐dependent alterations in gene expression. In line with this, MSCs harvested from abdominal subcutaneous fat of obese subjects exhibit epigenetic‐induced alterations in mitochondrial structure and function, associated with impaired fatty acid metabolism and neurogenic differentiation capacity [30]. Similarly, changes in the 5‐hydroxymethylcytosine profile of these cells are associated with amplified starvation‐induced reduction in autophagic flux and earlier generation of autophagosomes [110], suggesting that obesity also impairs autophagic homeostasis in human MSCs. Epigenetic and post‐transcriptional modifications have also been linked to changes in the expression of genes associated with apoptosis and senescence, inflammation [111, 112], and insulin resistance [113] in swine obese‐ and MetS‐MSCs.

Cardiovascular risk factors can also alter the cargo and impair the functionality of MSC‐derived EVs. Obesity attenuates the anti‐hypoxic, anti‐fibrotic, anti‐inflammatory, and tubular repair functions of human MSC‐derived EVs delivered in mice with chronic ischemic kidney disease [31]. Furthermore, obesity alters the size [114] and mRNA, miRNA, and protein cargo of MSC‐derived EVs, as well as their ability to modulate important injury pathways in recipient cells. Collectively, these changes impair MSC functional potency in vivo, thereby limiting the regenerative efficacy of MSC‐derived EVs.

Evidence regarding the effects of cardiovascular risk factors on tubular regenerative pathways remains comparatively limited and is currently derived primarily from experimental studies of swine STCs. Nevertheless, studies on swine STCs have shown that although the mitochondrial ultrastructure is relatively preserved, mitochondrial membrane potential and ATP production are reduced, whereas ROS production is increased in cells harvested from MetS pigs. Taken together, these observations suggest that the superimposition of cardiovascular risk factors magnifies renal ischemia‐induced changes in EPC‐mediated vascular repair, MSC function, and tubular regenerative pathways, thereby further compromising endogenous renal repair in patients with RVD.

### 2.6. Disease Stage and Phenotype Influence Endogenous Renal Repair

One important consideration is that RVD is not a uniform clinical entity, and the integrity and recoverability of endogenous repair mechanisms are likely to vary according to the disease stage, severity, and clinical phenotype [17, 19]. During the early stages of RVD, transient activation of the RAAS, increased expression of angiogenic mediators such as VEGF [47, 50] and SDF‐1 [46], and relatively preserved mitochondrial function may maintain the reparative capacity of endogenous repair cells, particularly EPCs [25]. As ischemia becomes chronic, however, persistent hypoxia, oxidative stress, inflammation, and microvascular rarefaction progressively affect EPC‐mediated vascular repair, MSC‐mediated stromal and paracrine support, and tubular regenerative pathways through mitochondrial dysfunction, cellular senescence, epigenetic remodeling, ER stress, and alterations in EV cargo. Consequently, endogenous repair may progressively shift from an adaptive regenerative response to irreversible cellular dysfunction and stem/progenitor cell exhaustion.

The clinical phenotype of RVD may further determine the likelihood of successful endogenous repair [19]. Patients with relatively preserved renal parenchyma, viable microvasculature, hemodynamically significant bilateral renal artery stenosis or a solitary functioning kidney, rapidly declining renal function, or recurrent flash pulmonary edema may retain sufficient endogenous repair capacity to benefit from interventions that restore renal perfusion [15–17]. In contrast, advanced disease characterized by extensive fibrosis, tubular atrophy, severe microvascular loss, and cellular senescence is less likely to recover despite successful revascularization, suggesting that regenerative therapies are likely to be less effective once endogenous repair mechanisms have become exhausted [19]. These observations support the concept of a therapeutic window during which preservation or restoration of endogenous repair systems is most likely to improve renal recovery before irreversible structural damage becomes established.

Future studies should therefore move beyond treating RVD as a single disease entity and instead define how the disease stage, duration of ischemia, degree of fibrosis, microvascular integrity, and molecular markers of stem/progenitor cell function influence therapeutic responsiveness. Such biomarkers may facilitate identification of patients in whom endogenous repair mechanisms remain recoverable, distinguish responder from non‐responder phenotypes, and guide the selection of medical therapy, renal revascularization, regenerative therapies, or combination approaches. This concept may also help explain the variable efficacy of renal revascularization and regenerative therapies reported in clinical studies as patients enrolled at advanced stages of RVD may have already exceeded the window during which endogenous repair mechanisms remain recoverable. Ultimately, integrating clinical phenotype with molecular biomarkers of endogenous repair may enable precision regenerative medicine by identifying the patients most likely to benefit from revascularization, cell‐based therapies, EV‐based approaches, or combination strategies.

### 2.7. Aging and Sex as Biological Variables

Aging is an important biological modifier of endogenous renal repair because RVD predominantly affects older adults, and several hallmarks of aging, including genomic instability, telomere attrition, epigenetic alterations, mitochondrial dysfunction, chronic inflammation, cellular senescence, and stem cell exhaustion, overlap mechanistically with RVD‐induced injury. In EPCs, advancing age is associated with lower circulating cell numbers, shorter telomeres, increased expression of senescence pathways, and diminished proliferation, migration, angiogenic activity, and vascular reparative capacity [115–117]. These age‐related abnormalities may further exacerbate RVD‐induced oxidative stress, nitric oxide depletion, and defective EPC mobilization [118], thereby promoting microvascular rarefaction. MSCs obtained from older donors similarly exhibit telomere shortening, DNA damage accumulation, mitochondrial dysfunction, oxidative stress, epigenetic drift, and activation of p16 and p53–p21 pathways, accompanied by reduced self‐renewal, differentiation, migration, immunomodulatory activity, and paracrine potency [119]. Consequently, aging may reduce the reparative efficacy of autologous MSCs while also altering the composition and biological activity of their secretome and EVs. Considerably less is known about age‐related changes across tubular regenerative pathways, including resident CD24^+^/CD133^+^ renal progenitor cells, STCs, and injury‐induced epithelial plasticity. Because tubular repair may involve resident progenitor populations, STCs, and transient plasticity of surviving epithelial cells, age‐associated telomere attrition, mitochondrial dysfunction, impaired proteostasis, and tubular cell senescence may compromise several components of this regenerative response. Resident renal progenitor cells and STCs isolated from young and aged kidneys have not been systematically compared, and the effect of aging on injury‐induced epithelial plasticity remains incompletely defined.

Sex is another important biological variable that may modify endogenous repair responses through sex chromosomes, gonadal hormones, and sex‐dependent differences in RAAS activity, vascular function, immunity, metabolism, and the response to renal ischemia. Experimental and clinical studies indicate that estrogen can increase EPC mobilization and telomerase activity and delay EPC senescence, whereas circulating EPC abundance and function decline after menopause, suggesting that age and sex interact in the regulation of vascular repair [120–123]. Biological sex may also influence MSC proliferation, differentiation, immunomodulation, and secretory activity, but reported findings vary according to tissue source, donor age, hormonal status, and culture conditions, and sex‐specific effects on kidney‐derived or RVD‐exposed MSCs remain poorly defined. Likewise, although male and female kidneys differ in susceptibility to ischemic injury and progression from AKI to fibrosis, whether these differences are mediated, at least in part, by sex‐dependent variation in the resident renal progenitor abundance, STC biology, epithelial plasticity, EV release, or tubular reparative potency remains unknown. The vasculature‐related mRNA and miRNA study by Kazeminia et al. [36–38] was conducted in female pigs, but it was not designed as a direct comparison between sexes and therefore cannot establish sexual dimorphism in STC responses to RVD. Future studies should include adequately powered male and female animals and cells, document donor sex, age, and hormonal status, analyze outcomes separately by sex, and test sex‐by‐age and sex‐by‐disease interactions.

### 2.8. EVs as Mediators of Endogenous Renal Repair

EVs have emerged as key mediators of stem/progenitor cell communication and represent one of the most promising cell‐free mediators of endogenous renal repair and therapeutic approaches for kidney disease. EVs comprise a heterogeneous population of membrane‐bound particles, including exosomes (30–150 nm), which originate from the endosomal pathway through inward budding of multivesicular bodies, and microvesicles (100–1000 nm), which are generated by outward budding of the plasma membrane [124–126]. Because EVs retain many of the molecular characteristics of their parent cells while avoiding several safety and manufacturing challenges associated with cell‐based therapies, they have attracted considerable interest as therapeutic agents for renal repair.

The biological activity of EVs is determined by their molecular cargo, which includes proteins, lipids, mRNAs, miRNAs, long non‐coding RNAs, DNA fragments, and mitochondrial components capable of modulating gene expression and cellular signaling in recipient cells [127, 128]. EVs are internalized through several complementary mechanisms, including receptor‐mediated endocytosis, clathrin‐ and caveolin‐dependent uptake, macropinocytosis, phagocytosis, and direct membrane fusion, allowing efficient horizontal transfer of bioactive molecules to target endothelial cells, tubular epithelial cells, macrophages, and other resident renal cells [129, 130]. As a result, EVs recapitulate many of the reparative properties of their parent stem/progenitor cells while serving as highly efficient mediators of intercellular communication.

Among endogenous renal repair systems, MSC‐derived EVs have been the most extensively characterized. MSCs exert many of their protective effects through paracrine signaling, including the secretion of cytokines, growth factors, and EVs enriched with mRNAs, miRNAs, and proteins capable of modulating several pathways responsible for renal injury [61, 62]. Swine adipose tissue‐derived MSCs actively secrete VEGF in the culture. Moreover, a single intra‐renal delivery of EVs derived from autologous adipose tissue‐derived MSCs improved medullary oxygenation, reduced fibrosis, and restored renal blood flow and GFR in pigs with renal artery stenosis [61]. miRNA‐ and mRNA‐sequencing analyses demonstrated that MSC‐derived EVs transport gene regulatory information involved in angiogenesis, adipogenesis, mitochondrial homeostasis, inflammation, and tissue remodeling [62]. Their cargo includes vasculoprotective genes that preserve the renal microvasculature, as well as mediators of TSG‐6 and TGF‐β signaling, which regulate macrophage polarization and promote regulatory T‐cell differentiation [63]. Importantly, the anti‐inflammatory effects of MSC‐derived EVs are partly attributed to their IL‐10 cargo as delivery of IL‐10‐silenced EVs failed to preserve the structure and function of stenotic swine kidneys [61], highlighting the critical contribution of specific EV cargo components to renal repair.

Emerging evidence indicates that STCs also exert important paracrine effects through EV secretion. Although research on STCs has traditionally focused on their capacity for direct tubular regeneration, recent studies demonstrate that the delivery of STC‐derived EVs improves renal perfusion and oxygenation and ameliorates inflammatory and profibrotic gene expression in ischemic murine kidneys. Similar to MSC‐derived EVs, STC‐derived vesicles appear capable of transferring regenerative signals to injured tubular epithelial cells, thereby enhancing endogenous repair while limiting inflammation and fibrosis. Nevertheless, the molecular composition of STC‐derived EVs remains considerably less characterized than that of MSC‐derived EVs, and additional studies are needed to define their complete RNA, protein, and lipid cargo as well as the signaling pathways responsible for their reparative effects. As with their parent stem/progenitor cells, the therapeutic efficacy of EVs is strongly influenced by the pathological microenvironment in which they are generated.

RVD and its associated cardiovascular risk factors substantially alter both the quantity and quality of reparative EVs. Proteomic analyses have shown that although healthy volunteer‐ and RVD‐derived MSCs released similar numbers and sizes of EVs, several proteins were dysregulated in EVs from RVD‐MSCs, including mitochondrial import proteins (e.g., TOMM7 and TIMM family members) and proteins involved in vesicle‐mediated transport, apoptosis, cell adhesion, and mitochondrial function [29]. These alterations parallel the mitochondrial dysfunction observed in RVD‐derived MSCs and likely contribute to the reduced reparative potency of their EVs. In addition, RVD‐induced changes in cellular miRNA expression are expected to modify the EV cargo, although this area remains incompletely understood.

Cardiovascular risk factors further compromise EV biology. Obesity attenuates the anti‐hypoxic, anti‐inflammatory, anti‐fibrotic, and tubular reparative effects of human MSC‐derived EVs in experimental chronic ischemic kidney disease [31]. In addition, obesity and MetS alter EV biogenesis by changing EV size distribution [114] and modify their molecular cargo, including mRNA, miRNA, and protein composition, thereby impairing their ability to regulate angiogenesis, mitochondrial homeostasis, inflammation, and tissue repair in recipient cells. In particular, MetS enriches EVs with senescence‐associated and mitochondria‐related miRNAs while altering the packaging of proteins involved in the mitochondrial function and cellular metabolism. Collectively, these molecular changes diminish the biological activity of MSC‐derived EVs and limit their ability to restore the renal structure and function in vivo. Comparable alterations in STC‐derived EVs have not yet been systematically investigated, representing an important knowledge gap in the field.

Taken together, current evidence suggests that EVs represent a central mechanism by which MSCs and at least some cellular components of tubular regenerative pathways, particularly STCs, contribute to endogenous renal repair. Their ability to deliver complex combinations of regulatory RNAs, proteins, lipids, and immunomodulatory mediators to injured renal cells makes them attractive therapeutic candidates for RVD. However, the efficacy of EV‐based therapies depends critically on the preservation of EV biogenesis and cargo composition, both of which are compromised by renal ischemia and cardiovascular comorbidities such as obesity, diabetes, and MetS. A better understanding of how disease modifies EV production, molecular composition, tissue targeting, and cellular uptake may facilitate the development of engineered or preconditioned EVs with enhanced regenerative potential, thereby accelerating the clinical translation of cell‐free regenerative therapies for RVD and other forms of kidney disease.

### 2.9. Novel Therapeutic Strategies

Therapeutic approaches to preserve endogenous renal repair can be broadly divided into two complementary strategies: (1) in vivo interventions, which aim to preserve or restore the function of endogenous repair cells within the ischemic kidney by targeting the molecular pathways responsible for their dysfunction and (2) ex vivo manipulation, in which stem/progenitor cells are modified before therapeutic administration to enhance their reparative potency (Figure 2). Although both approaches ultimately aim to preserve or restore endogenous renal repair, they differ substantially in their mechanisms of action, translational challenges, and clinical applicability.

#### 2.9.1. Strategies to Preserve Endogenous Repair In Vivo

Several targeted interventions have been developed to preserve endogenous vascular, stromal, and tubular repair mechanisms by directly targeting mitochondrial dysfunction, ER stress, epigenetic alterations, and post‐transcriptional dysregulation. For example, co‐incubation of swine RVD‐STCs with the mitochondria‐targeted peptide SS‐31, which stabilizes mitochondrial inner membrane cardiolipin [131], preserved mitochondrial structure and membrane potential, reduced ROS production, and enhanced the ability of these cells to repair injured tubular epithelial cells in vitro [33]. Similarly, treatment of swine RVD‐STCs with sodium hydrogen sulfide (NaHS), an ER‐stress modulator, decreased expression of ER stress markers and ER dilation and improved the ability of these cells to protect injured human tubular epithelial cells in vitro [38], suggesting that preservation of proteostasis may enhance endogenous tubular repair. However, whether ER stress modulation improves the reparative capacity of STCs in vivo remains to be established.

Epigenetic and post‐transcriptional regulation also represent promising therapeutic targets. Treatment with the epigenetic modulator vitamin C reversed global epigenetic changes, normalized the expression of apoptosis‐ and senescence‐related genes, and preserved mitochondrial structure and function in swine MetS‐MSCs. Similarly, epigenetic modulation ameliorated mitochondrial structural abnormalities and improved neuronal differentiation of human adipose‐derived MSCs harvested from obese individuals [30] while normalizing inflammatory gene and protein expression in RVD‐STCs [36]. Collectively, these findings suggest that pharmacological modulation of epigenetic and stress‐response pathways may preserve the reparative capacity of endogenous stem/progenitor cells without requiring cell isolation or transplantation.

#### 2.9.2. Ex Vivo Enhancement of Stem/Progenitor Cell Therapies

A complementary strategy aims to improve the reparative potential of stem/progenitor cells before therapeutic administration through ex vivo manipulation. These approaches seek to enhance cell survival, mitochondrial function, angiogenic activity, and paracrine signaling prior to transplantation, thereby increasing the resistance to the hostile ischemic microenvironment.

For example, co‐incubation of human MSCs with an antagomir against miR‐378h, which targets several mitochondria‐related genes, improved mitochondrial structure and reparative function both in vitro and in vivo [29], further supporting mitochondria as a central therapeutic target for enhancing stem/progenitor cell potency. Similarly, treatment of swine RVD‐STCs with modulators of miR‐146a‐3p and miR‐17‐3p, which regulate vasculature‐related genes, decreased the expression of anti‐angiogenic genes while increasing the expression of proangiogenic targets [37]. This intervention partially restored the proangiogenic phenotype of RVD‐STCs in vitro, as evidenced by improved tubular‐like structure formation on Matrigel, suggesting that post‐transcriptional regulation contributes to RVD‐induced stem/progenitor cell dysfunction. However, these findings were limited to an in vitro angiogenesis assay, and whether miRNA modulation improves STC survival, engraftment, or reparative function after transplantation remains unknown. Accordingly, additional preclinical studies are needed to establish the efficacy, safety, and translational potential of ex vivo miRNA‐based enhancement strategies.

Hypoxia preconditioning (HPC) is another promising ex vivo approach designed to optimize the therapeutic effectiveness of MSCs before administration. Experimental studies in swine RVD have shown that HPC augments the regenerative capacity of adipose tissue‐derived MSCs partly by mitigating pathological molecular effects on inflammatory and profibrotic genes [132]. However, studies in humans demonstrated a more favorable functional effect in MSCs harvested from healthy kidney donors than in those obtained from patients with hypertensive kidney disease. In addition, HPC also induced epigenetic changes that enhanced angiogenic gene expression while suppressing senescence pathways in swine MSCs, underscoring the potential of ex vivo preconditioning to improve therapeutic cell quality.

Collectively, these studies suggest that both in vivo and ex vivo strategies have considerable potential to preserve or restore endogenous renal repair. In vivo therapies directly target the pathological microenvironment responsible for stem/progenitor cell dysfunction and may preserve endogenous repair mechanisms without requiring cell harvesting or expansion. In contrast, ex vivo manipulation offers greater control over cell quality and potency before transplantation but requires cell isolation, manufacturing, quality control, and regulatory oversight. These approaches should therefore be viewed as complementary rather than competing therapeutic paradigms.

Despite encouraging preclinical results, important translational challenges remain. Generation of autologous EPCs requires isolation of peripheral blood mononuclear cells, followed by several weeks of ex vivo expansion in endothelial media, necessitating large blood volumes from each patient [133]. The therapeutic use of tubular reparative cells presents additional challenges. STCs constitute only a small fraction of dissociated kidney cells [33], whereas resident human renal progenitor cells generally require kidney tissue procurement and ex vivo expansion. Consequently, biopsy requirements, low cell yield, and uncertain population identity remain important translational barriers. Although MSCs are more readily accessible from adipose tissue, bone marrow, or umbilical cord tissue, concerns remain regarding long‐term safety, including rare reports of sarcoma and angiomyeloproliferative lesions following cell therapy [134, 135]. Furthermore, ex vivo manipulation introduces additional challenges related to manufacturing, standardization, batch‐to‐batch variability, quality control, potency testing, scalability, and regulatory approval. Future studies should identify the patients most likely to benefit from preservation of endogenous repair in vivo versus administration of ex vivo‐enhanced therapeutic cells, establish biomarkers that predict therapeutic responsiveness, and determine whether combining these complementary strategies provides additive or synergistic renoprotective effects.

### 2.10. Clinical Translation of MSC Therapy for Renal Repair

Clinical translation of MSC‐based therapy for kidney disease remains in its early stages and is currently supported by only a small number of predominantly phase 1 and phase 2 studies (Table 2). The most directly relevant evidence for RVD derives from a phase 1 dose‐escalation study of intra‐arterial autologous adipose‐derived MSCs in patients with atherosclerotic renal artery stenosis. Initial results from 14 treated patients demonstrated that intra‐arterial MSC administration was well tolerated and was associated with increased cortical perfusion and renal blood flow, as well as reduced tissue hypoxia after 3 months [21]. Subsequent expansion to 21 treated patients across three dose cohorts demonstrated modest improvements in measured GFR and renal blood flow, reductions in blood pressure and inflammatory biomarkers, and no major cell‐related toxicity. Nevertheless, this study was nonrandomized, involved small dose cohorts, and was primarily designed to assess safety; therefore, these encouraging efficacy signals require confirmation in adequately powered randomized trials. Trials in diabetic kidney disease have likewise demonstrated the short‐term feasibility and safety of intravenous allogeneic mesenchymal lineage cells, with preliminary evidence suggesting stabilization of GFR in some studies, whereas a larger phase 2 trial in cardiac surgery‐associated AKI failed to accelerate kidney recovery. Overall, differences in kidney disease etiology, MSC source, autologous versus allogeneic administration, dose, route, timing, and outcome assessment currently preclude definitive conclusions regarding the clinical efficacy of MSC therapy across kidney diseases. Collectively, these early clinical studies support the feasibility and short‐term safety of MSC therapy in patients with RVD, but larger randomized controlled trials are needed to determine whether improvements in renal perfusion translate into durable preservation of kidney function and improved clinical outcomes. Future clinical trials should also consider patient selection, disease stage, and the integrity of endogenous repair mechanisms as these factors are likely to influence responsiveness to regenerative therapies.

**Table 2:** Selected clinical trials of MSC‐based therapy for renal repair.

Kidney condition	Cell type	Phase/design	Route and dose	*n*	Primary endpoint	Key result or current status	ClinicalTrials.gov ID
Atherosclerotic RVD	Autologous adipose‐derived MSCs	Phase 1a, open‐label, nonrandomized dose escalation with matched medically treated controls	Single infusion into the stenotic renal artery; 1.0, 2.5, or 5.0 × 10^5^ cells/kg	39 total: 21 MSC‐treated and 18 controls	Safety and tolerability; renal hemodynamic and functional measures	Completed. Well tolerated; increased stenotic‐kidney blood flow, modestly increased measured GFR, reduced renal hypoxia, inflammatory biomarkers, and systolic blood pressure at 3 months. Saad et al. reported the first 14 treated patients; Abumoawad et al. reported the expanded cohort.	NCT01840540
Diabetic kidney disease	Allogeneic bone marrow‐derived mesenchymal precursor cells, rexlemestrocel‐L	Phase 1/2, randomized, double‐blind, placebo‐controlled dose escalation	Single IV infusion; 150 × 10^6^ or 300 × 10^6^ cells	30	Safety and tolerability through 60 weeks	Completed. No treatment‐related serious adverse events or persistent donor‐specific sensitization; exploratory analyses suggested stabilization or improvement of eGFR and measured GFR, but the study was not powered for efficacy.	NCT01843387
Progressive diabetic kidney disease—NEPHSTROM	Allogeneic bone marrow‐derived, anti‐CD362‐selected MSCs, ORBCEL‐M	Phase 1b/2a, randomized, placebo‐controlled dose escalation	Single IV infusion; published first cohort received 80 × 10^6^ cells	16 in the published low‐dose cohort; larger planned study incompletely enrolled	Safety and tolerability; preliminary efficacy based on measured and estimated GFR	Terminated after partial enrollment. The low‐dose cohort was well tolerated. Decline in eGFR was slower with ORBCEL‐M, but measured GFR did not differ; the registry reports termination because the investigational product expired and recruitment was low at one center.	NCT02585622
Cardiac surgery‐associated AKI—ACT‐AKI	Allogeneic bone marrow‐derived MSCs, AC607	Phase 2, multicenter, randomized, double‐blind, placebo‐controlled	Single intra‐aortic infusion proximal to the renal arteries; 2 × 10^6^ cells/kg	156 randomized; 135 dosed	Time to recovery of kidney function, defined by return of serum creatinine to baseline	Terminated/completed analysis. MSCs did not shorten kidney recovery: median 15 days with MSCs versus 12 days with placebo. Mortality, dialysis requirement, and adverse‐event rates were not significantly different.	NCT01602328
Trauma‐associated stage 2 AKI	Allogeneic adipose‐derived MSCs, HB‐adMSCs	Phase 1/2a, multicenter, randomized, double‐blind, placebo‐controlled	IV infusion; dosing specified in the active protocol	Estimated enrollment 50	Safety and prevention of progression of trauma‐associated AKI	Recruiting as of July 2026. No efficacy results available. The study includes patients with AKI after major trauma, burns, or crush injury.	NCT06654193
AKI—Amimestrocel	Allogeneic human umbilical cord‐derived MSCs	Phase 2, open‐label, single‐group	IV infusion of 1 × 10^6^ cells/kg on days 1, 4, 8, and 15	Estimated enrollment 50	Change in serum creatinine at day 28	Ongoing; enrollment began in June 2025. Estimated primary completion in May 2026 and study completion in 2028; no results posted.	NCT06954740
Stage 3–4 CKD, including diabetic and hypertensive kidney disease	Allogeneic umbilical cord tissue‐derived MSCs	Phase 1/2, randomized, placebo‐controlled	IV infusion; protocol‐defined MSC regimen	Estimated enrollment 32	Changes in eGFR, 24‐h proteinuria, and urinary albumin‐to‐creatinine ratio through 12 months	Not yet recruiting in the most recently posted registry record. Planned comparison with saline placebo; no results available.	NCT07240987

*Note*: Status was obtained from ClinicalTrials.gov and verified in July 2026. “*n*” indicates actual enrollment for completed studies and estimated enrollment for ongoing studies.

Abbreviations: AKI, acute kidney injury; CKD, chronic kidney disease; DKD, diabetic kidney disease; GFR, glomerular filtration rate; IV, intravenous; MSC, mesenchymal stromal/stem cell; RVD, renovascular disease.

### 2.11. Emerging Regenerative Cell Platforms: Induced Pluripotent Stem Cells (iPSCs) and Kidney Organoids

Although endogenous EPCs, MSCs, and cellular components of tubular regenerative pathways possess considerable reparative potential, their clinical translation is limited by low cell abundance, the need for invasive tissue procurement or prolonged ex vivo expansion, donor‐to‐donor variability, and functional impairment associated with aging, RVD, and cardiovascular comorbidities. These limitations have stimulated interest in alternative regenerative platforms, including iPSCs, kidney organoids, and iPSC‐derived vascular and mesenchymal cells, which offer potentially unlimited sources of standardized therapeutic cells. Unlike adult stem/progenitor cells, iPSCs are generated by reprograming differentiated somatic cells into a pluripotent state through the expression of defined transcription factors [136, 137]. Consequently, they retain the capacity for unlimited self‐renewal and can be differentiated into virtually any renal or vascular cell type, including endothelial cells, pericyte‐like mesenchymal cells, podocytes, and tubular epithelial cells. These characteristics make iPSC‐derived products particularly attractive for kidney regenerative medicine, where limited cell availability and impaired autologous cell function remain major barriers to clinical translation.

Unlike strategies aimed at preserving or enhancing endogenous adult stem/progenitor cells, iPSC‐based platforms offer the possibility of generating standardized cell products that are largely independent of donor age, disease status, and tissue availability. Significant advances in developmental biology have enabled the generation of three‐dimensional kidney organoids from human iPSCs that recapitulate many structural and transcriptional features of the developing nephron, including podocytes, proximal and distal tubular epithelial cells, collecting duct‐like cells, and stromal populations [138, 139]. Although current organoids remain immature and lack a fully functional vascular network, they have become valuable platforms for studying kidney development, disease mechanisms, nephrotoxicity, and regenerative pathways. In addition, iPSC‐derived endothelial cells exhibit robust angiogenic potential and have been shown to improve microvascular repair and tissue perfusion in experimental models of ischemic injury, whereas iPSC‐derived mesenchymal stromal cells retain many of the immunomodulatory and paracrine properties of adult MSCs while exhibiting greater proliferative capacity and more consistent manufacturing characteristics [140]. EVs derived from iPSC‐derived MSCs also exhibit potent anti‐inflammatory, anti‐fibrotic, and proangiogenic activities in experimental kidney injury, suggesting that cell‐free approaches may further reduce concerns regarding cell persistence and tumorigenicity [141].

Despite these promising advances, several challenges remain before iPSC‐based therapies can be applied to patients with RVD. Residual undifferentiated iPSCs may give rise to teratomas, necessitating rigorous purification and quality control procedures prior to clinical use. Furthermore, genetic and epigenetic abnormalities acquired during reprograming or prolonged culture, incomplete cellular maturation, immunogenicity of allogeneic products, and large‐scale manufacturing under good manufacturing practice (GMP) conditions remain important obstacles. Kidney organoids also require improved vascularization, maturation, and integration with the host tissue before they can be considered for therapeutic implantation. Consequently, current efforts are increasingly focused on generating defined populations of differentiated iPSC‐derived endothelial cells, MSCs, and renal epithelial cells rather than transplanting pluripotent cells themselves.

Although no studies have yet investigated iPSC‐derived regenerative therapies specifically in experimental or clinical RVD, these emerging technologies have the potential to overcome several important limitations associated with autologous EPC‐, MSC‐, and tubular progenitor/STC‐based therapies. Standardized iPSC‐derived cell products could provide an “off‐the‐shelf” source of reparative cells with consistent potency, while gene editing, preconditioning strategies, and bioengineering approaches may further enhance the resistance to ischemia, oxidative stress, and inflammation. Future studies should evaluate whether iPSC‐derived vascular and renal cell populations can restore microvascular integrity, preserve endogenous repair mechanisms, and improve renal function in ischemic kidney diseases such as RVD while establishing their long‐term safety, durability, and therapeutic efficacy.

### 2.12. Safety, Manufacturing, and Regulatory Considerations

Despite encouraging safety findings in early‐phase kidney trials, several issues must be addressed before MSC‐based therapies can be broadly translated into clinical practice. Short‐term studies have generally reported that MSC administration is feasible and well tolerated; however, small cohorts, heterogeneous products, and limited follow‐up preclude definitive conclusions regarding uncommon or delayed adverse events. The safety profile of MSC therapy is influenced by multiple interacting variables, including whether the product is autologous or allogeneic, the tissue and donor source, culture duration, passage number, cryopreservation and thawing procedures, dose, route of administration, and the recipient’s underlying disease and immune status.

Tumorigenicity remains an important theoretical and regulatory concern. Unlike pluripotent stem cells, adult MSCs do not ordinarily form teratomas, and convincing evidence that appropriately manufactured human MSC products directly generate malignant tumors is limited. Nevertheless, prolonged ex vivo expansion can promote replicative senescence, chromosomal instability, or the selection of abnormal clones, particularly when cells are extensively passaged or manufactured under poorly controlled conditions. Case reports of sarcoma or angiomyeloproliferative lesions following cell therapy have raised concern, although the identity of the administered cells and a direct causal relationship were not always firmly established [134, 135]. MSCs may also exert indirect protumorigenic effects by promoting angiogenesis, modifying immune surveillance, or supporting the tumor‐associated stroma. Clinical‐grade products should therefore undergo testing for identity, purity, viability, sterility, genomic stability, and evidence of inappropriate differentiation or transformation, with limits placed on the passage number and culture duration. Long‐term surveillance is particularly important when cells persist in tissues or have been genetically or extensively manipulated.

Immunogenicity is also relevant, especially for allogeneic products. MSCs express relatively low basal levels of major histocompatibility complex class II and costimulatory molecules and possess immunomodulatory properties, but they should not be considered immunologically invisible. Inflammatory stimulation can increase human leukocyte antigen (HLA) expression, and repeated or high‐dose allogeneic administration may induce cellular immune responses, donor‐specific antibodies, complement activation, or accelerated clearance. The clinical consequences of alloimmunization remain incompletely defined but may be particularly relevant for patients who subsequently require kidney transplantation. Donor screening, HLA and antibody monitoring, assessment of pre‐existing sensitization, and longer follow‐up after repeated administration should therefore be incorporated into clinical protocols. Although autologous products avoid donor‐recipient alloimmunity, their therapeutic potency may be compromised because aging, RVD, diabetes, obesity, and MetS adversely alter MSC mitochondrial function, senescence, secretory activity, and EV cargo.

The route and dose of administration introduce additional safety considerations. Intravenously infused MSCs are relatively large and are initially retained within the pulmonary microcirculation, creating a potential risk of transient pulmonary vascular obstruction, complement and coagulation activation, or thromboembolic complications. Targeted intra‐arterial delivery may increase cell retention within the kidney and reduce systemic exposure but can produce renal microvascular obstruction if the cell concentration, infusion rate, or total dose is excessive. In a porcine study, intrarenal arterial administration of 10 million MSCs was well tolerated, whereas administration of 100 million cells caused an acute reduction in renal perfusion, glomerular microthrombi, inflammation, and subsequent glomerular and tubular injury, demonstrating a dose‐dependent safety threshold [142]. These observations support careful dose escalation, slow infusion under controlled conditions, avoidance of cell aggregation, standardized assessment of cell size and viability, and close monitoring for renal infarction, thrombosis, acute GFR decline, pulmonary complications, and instant blood‐mediated inflammatory reactions. This issue is particularly relevant in RVD, where an already narrowed renal artery and compromised downstream microcirculation may increase the susceptibility to embolic or obstructive injury.

Product heterogeneity and batch‐to‐batch variability are major obstacles to reproducibility and safety. MSC phenotype and potency vary with donor age, sex, comorbidities, tissue source, isolation technique, culture medium, oxygen tension, passage number, cell density, senescence burden, cryopreservation, and post‐thaw recovery [143, 144]. These factors can alter proliferation, immunomodulation, angiogenesis, mitochondrial function, tissue factor expression, and the composition of the secretome and EVs [143, 144]. Reliance on surface‐marker identity and trilineage differentiation alone is therefore insufficient to ensure therapeutic equivalence [145]. Manufacturing should comply with current GMP standards and include predefined release criteria for identity, purity, viability, sterility, endotoxin and mycoplasma contamination, adventitious agents, genomic stability, aggregation, and procoagulant activity [145]. Because no single assay captures the pleiotropic mechanisms of MSCs, a panel of mechanism‐based potency assays, such as suppression of inflammatory cell activity, macrophage polarization, angiogenic support, and secretion of defined reparative factors, may be needed to establish comparability between donors, batches, manufacturing changes, and clinical sites [145].

Regulatory classification depends on the degree of manipulation and the intended use. In the United States, expanded MSCs intended to treat kidney disease generally do not meet the criteria for minimally manipulated, homologously used human cells, tissues, and cellular and tissue‐based products and are therefore regulated as biological products requiring an Investigational New Drug application and, ultimately, premarket biologics approval. Development programs must define active substances, manufacturing process, critical quality attributes, potency, biodistribution, persistence, tumorigenicity, immunogenicity, and dose‐ and route‐specific toxicology. In the European Union, most expanded MSC products are classified as advanced therapy medicinal products and are subject to corresponding quality, nonclinical, clinical, pharmacovigilance, and risk management requirements. Current regulatory guidance emphasizes traceability, donor eligibility, validated manufacturing and release testing, comparability after process changes, and long‐term follow‐up tailored to the persistence and biological activity of the product. Unregulated stem‐cell interventions administered outside authorized trials should be clearly distinguished from rigorously manufactured investigational MSC products.

Cell‐free products such as MSC‐derived EVs may reduce concerns related to uncontrolled proliferation, ectopic differentiation, persistent engraftment, and microvascular obstruction by intact cells, although they are not without potential risks. EV preparations may vary in particle composition, potency, purity, biodistribution, and procoagulant or immunologic activity and may co‐isolate proteins, lipoproteins, nucleic acids, or process‐related contaminants. Standardized definitions, scalable GMP production, validated purification and storage methods, dose metrics, potency assays, and biodistribution and toxicity studies will be required before EV‐based therapies can be considered safer or more reproducible alternatives to MSC administration.

Overall, current evidence supports a favorable short‐term safety profile for carefully manufactured MSC products administered at conservative doses, but it does not establish long‐term safety or efficacy. Future renal trials should incorporate prospective monitoring for malignancy, alloimmunization, thromboembolism, microvascular obstruction, infection, ectopic tissue formation, and deterioration of renal or pulmonary function. Standardized manufacturing, mechanism‐linked potency testing, transparent adverse event reporting, and prolonged follow‐up will be essential to define the therapeutic window, facilitate meaningful comparison across studies, and support the safe and effective clinical translation of MSC‐based therapies.

## 3. Conclusion

RVD remains an important cause of secondary hypertension and renal dysfunction in the elderly population and is associated with increased cardiovascular morbidity and mortality. Emerging evidence demonstrates that vascular repair mediated by EPCs, stromal and paracrine support mediated by MSCs, and tubular regenerative pathways constitute complementary components of endogenous kidney repair. Tubular regenerative pathways include resident renal progenitor cells, STCs, and injury‐induced epithelial plasticity, which should be regarded as related but biologically distinct concepts. Preclinical studies have consistently shown that exogenous administration of EPCs, MSCs, renal progenitor cells, or experimentally isolated STCs attenuates structural kidney injury, improves renal perfusion and function, and limits fibrosis. Their reparative effects are mediated through distinct yet complementary mechanisms, including angiogenesis, immunomodulation, tubular regeneration, and paracrine signaling. Potential synergistic interactions among these cell populations remain largely unexplored and represent an important area for future investigation. For example, both MSCs and experimentally isolated tubular reparative cells promote endothelial repair and angiogenesis, but whether they directly enhance EPC proliferation, mobilization, or function remains unknown. Similarly, although inflammation impairs the reparative capacity of STCs, it is unclear whether the anti‐inflammatory properties of MSCs or their EVs can preserve the STC function in the setting of RVD.

RVD adversely affects endogenous repair systems, although the magnitude and timing of these effects differ among cell populations. The reparative capacity of MSCs and experimentally characterized STCs is consistently impaired in RVD, whereas the effects of RVD on the resident renal progenitor cells and injury‐induced epithelial plasticity remain less well defined. In contrast, the effects of RVD on EPCs appear to be more dynamic. Experimental studies suggest that EPC angiogenic activity is initially preserved or even transiently enhanced during the early stages of RVD, likely reflecting the compensatory activation of RAAS and increased VEGF‐ and SDF‐1‐mediated mobilization. Similarly, although circulating EPC numbers are reduced in patients with medically treated RVD, their migratory, proliferative, and tube‐forming capacities remain largely preserved, suggesting that contemporary medical therapy may maintain the EPC function despite diminished cell availability. Whether prolonged disease duration, persistent oxidative stress, inflammation, or progressive renal injury ultimately result in clinically meaningful EPC dysfunction remains unclear and warrants further investigation.

The mechanisms underlying RVD‐induced stem/progenitor cell dysfunction converge on common intracellular pathways, including mitochondrial dysfunction, oxidative stress, epigenetic remodeling, miRNA dysregulation, ER stress, and cellular senescence, which collectively compromise endogenous renal repair. These processes are further exacerbated by cardiovascular risk factors such as obesity, diabetes, and MetS, emphasizing the importance of early intervention and comprehensive cardiovascular risk reduction to preserve endogenous repair mechanisms.

Therapeutic strategies aimed at restoring endogenous repair, including mitoprotective agents, epigenetic modifiers, miRNA‐targeted therapies, HPC, and EV‐based approaches, have shown encouraging results in experimental models. However, several important challenges must be addressed before regenerative therapies can be translated into routine clinical practice. First, standardized protocols for the isolation, characterization, expansion, and potency assessment of EPCs, MSCs, resident renal progenitor cells, STCs, and their EVs are needed to improve reproducibility and facilitate comparison across studies. Harmonization of cell identity markers, functional assays, and manufacturing criteria will be essential for regulatory approval and clinical implementation.

Second, future preclinical and clinical studies should move beyond evaluating individual cell populations in isolation and instead perform head‐to‐head comparisons of EPCs, MSCs, resident renal progenitor cells, experimentally defined STCs, and their EVs using standardized experimental models and clinically relevant outcome measures. Such studies should also investigate whether combinatorial strategies provide additive or synergistic benefits compared with single‐cell approaches.

Third, greater emphasis should be placed on precision regenerative medicine by identifying biomarkers that predict therapeutic responsiveness. Molecular signatures reflecting mitochondrial integrity, cellular senescence, inflammatory status, epigenetic modifications, miRNA profiles, and EV cargo composition may help distinguish responder from non‐responder phenotypes and guide patient selection for autologous versus allogeneic therapies.

Finally, emerging regenerative platforms, including engineered EVs, iPSC‐derived endothelial, mesenchymal, and renal epithelial cells, and kidney organoids, offer the potential to overcome limitations associated with harvesting and expansion of endogenous repair cells while providing scalable, standardized therapeutic products. Determining whether these next‐generation approaches can preserve microvascular integrity, enhance endogenous renal repair, and improve long‐term clinical outcomes in patients with RVD represents an important priority for future investigation.

Ultimately, integrating advances in stem/progenitor cell biology, EV therapeutics, bioengineering, biomarker discovery, and precision regenerative medicine with optimized medical therapy and selective renal revascularization offers the opportunity to preserve endogenous renal repair and develop personalized regenerative strategies capable of slowing the progression of ischemic kidney injury in patients with RVD.

## Author Contributions

Vinaya C. Iyer prepared the first draft of the manuscript. Lilach O. Lerman and Alfonso Eirin contributed to manuscript review, critical revision, and editing.

## Funding

This work was supported by the National Institutes of Health grants DK129240 and HL158691 and Regenerative Medicine Minnesota (Grant RMM 091620 DS 004).

## Disclosure

The final version of the manuscript was approved by all the authors.

## Conflicts of Interest

The authors declare no conflicts of interest.

## Data Availability

Data sharing is not applicable to this article as no datasets were generated or analyzed during the current study.

## References

[1] Safian R. D. and Textor S. C. , Renal-Artery Stenosis, The New England Journal of Medicine. (2001) 344, no. 6, 431–442, 10.1056/NEJM200102083440607. 11172181

[2] Hansen K. J. , Edwards M. S. , and Craven T. E. , et al.Prevalence of Renovascular Disease in the Elderly: A Population-Based Study, Journal of Vascular Surgery. (2002) 36, no. 3, 443–451, 10.1067/mva.2002.127351. 12218965

[3] Coen G. , Manni M. , and Giannoni M. F. , et al.Ischemic Nephropathy in an Elderly Nephrologic and Hypertensive Population, American Journal of Nephrology. (1998) 18, no. 3, 221–227, 10.1159/000013340. 9627038

[4] Kalra P. A. , Guo H. , and Kausz A. T. , et al.Atherosclerotic Renovascular Disease in United States Patients Aged 67 Years or Older: Risk Factors, Revascularization, and Prognosis, Kidney International. (2005) 68, no. 1, 293–301, 10.1111/j.1523-1755.2005.00406.x. 15954920

[5] Leertouwer T. C. , Pattynama P. M. T. , and Van Den Berg-Huysmans A. , Incidental Renal Artery Stenosis in Peripheral Vascular Disease: A Case for Treatment?, Kidney International. (2001) 59, no. 4, 1480–1483, 10.1046/j.1523-1755.2001.0590041480.x. 11260411

[6] Harding M. B. , Smith L. R. , and Himmelstein S. I. , et al.Renal Artery Stenosis: Prevalence and Associated Risk Factors in Patients Undergoing Routine Cardiac Catheterization, Journal of the American Society of Nephrology. (1992) 2, no. 11, 1608–1616, 10.1681/ASN.V2111608. 1610982

[7] Saran R. , Robinson B. , and Abbott K. C. , et al.US Renal Data System 2016 Annual Data Report: Epidemiology of Kidney Disease in the United States, American Journal of Kidney Diseases. (2017) 69, no. 3, A7–A8, 10.1053/j.ajkd.2016.12.004. PMC6605045 28236831

[8] Fatica R. A. , Port F. K. , and Young E. W. , Incidence Trends and Mortality in End-Stage Renal Disease Attributed to Renovascular Disease in the United States, American Journal of Kidney Diseases. (2001) 37, no. 6, 1184–1190, 10.1053/ajkd.2001.24521. 11382687

[9] Conlon P. J. , Little M. A. , Pieper K. , and Mark D. B. , Severity of Renal Vascular Disease Predicts Mortality in Patients Undergoing Coronary Angiography, Kidney International. (2001) 60, no. 4, 1490–1497, 10.1046/j.1523-1755.2001.00953.x. 11576364

[10] Pelta A. , Andersen U. B. , Just S. , and Bækgaard N. , Flash Pulmonary Edema in Patients With Renal Artery Stenosis—The Pickering Syndrome, Blood Pressure. (2011) 20, no. 1, 15–19, 10.3109/08037051.2010.527445. 21034349

[11] Pillay W. R. , Kan Y. M. , Crinnion J. N. , and Wolfe J. H. N. , Prospective Multicentre Study of the Natural History of Atherosclerotic Renal Artery Stenosis in Patients With Peripheral Vascular Disease, British Journal of Surgery. (2002) 89, no. 6, 737–740, 10.1046/j.1365-2168.2002.02144.x. 12027983

[12] Hackam D. G. , Duong-Hua M. L. , and Mamdani M. , et al.Angiotensin Inhibition in Renovascular Disease: A Population-Based Cohort Study, American Heart Journal. (2008) 156, no. 3, 549–555, 10.1016/j.ahj.2008.05.013. 18760140

[13] Chrysochou C. , Foley R. N. , Young J. F. , Khavandi K. , Cheung C. M. , and Kalra P. A. , Dispelling the Myth: The use of Renin-Angiotensin Blockade in Atheromatous Renovascular Disease, Nephrology, Dialysis, Transplantation. (2012) 27, no. 4, 1403–1409, 10.1093/ndt/gfr496. 21993376

[14] Cheung C. M. , Patel A. , and Shaheen N. , et al.The Effects of Statins on the Progression of Atherosclerotic Renovascular Disease, Nephron Clinical Practice. (2007) 107, no. 2, c35–c42, 10.1159/000107552. 17713349

[15] Investigators A. , et al.Revascularization Versus Medical Therapy for Renal-Artery Stenosis, The New England Journal of Medicine. (2009) 361, no. 20, 1953–1962, 10.1056/NEJMoa0905368. 19907042

[16] Cooper C. J. , Murphy T. P. , and Cutlip D. E. , et al.Stenting and Medical Therapy for Atherosclerotic Renal-Artery Stenosis, New England Journal of Medicine. (2014) 370, no. 1, 13–22, 10.1056/NEJMoa1310753. PMC4815927 24245566

[17] Textor S. C. , Misra S. , and Oderich G. S. , Percutaneous Revascularization for Ischemic Nephropathy: The Past, Present, and Future, Kidney International. (2013) 83, no. 1, 28–40, 10.1038/ki.2012.363. PMC3532568 23151953

[18] Textor S. C. , Renovascular Hypertension: Is There Still a Role for Stent Revascularization?, Current Opinion in Nephrology and Hypertension. (2013) 22, no. 5, 525–530, 10.1097/MNH.0b013e328363ffe0. PMC4263252 23917028

[19] Eirin A. , Chade A. R. , and Lerman L. O. , Kidney Intrinsic Mechanisms as Novel Targets in Renovascular Hypertension, Hypertension. (2024) 81, no. 2, 206–217, 10.1161/HYPERTENSIONAHA.123.21362. PMC10842320 37869904

[20] Botros F. T. , Olszanecki R. , Prieto-Carrasquero M. C. , Goodman A. I. , Navar L. G. , and Abraham N. G. , Induction of Heme Oxygenase-1 in Renovascular Hypertension Is Associated With Inhibition of Apoptosis, Cellular and Molecular Biology. (2007) 53, no. 4, 51–60. 17531161

[21] Saad A. , Dietz A. B. , and Herrmann S. M. S. , et al.Autologous Mesenchymal Stem Cells Increase Cortical Perfusion in Renovascular Disease, Journal of the American Society of Nephrology. (2017) 28, no. 9, 2777–2785, 10.1681/ASN.2017020151. PMC5576946 28461553

[22] Abumoawad A. , Saad A. , and Ferguson C. M , et al.In a Phase 1a Escalating Clinical Trial, Autologous Mesenchymal Stemcell Infusion for Renovascular Disease Increases Blood Flow and the Glomerular Filtration Rate While Reducing Inflammatory Biomarkers and Blood Pressure, Kidney International. (2020) 97, no. 4, 793–804. 10.1016/j.kint.2019.11.022 PMC7284953 32093917

[23] Chade A. R. , Zhu X.-Y. , and Krier J. D. , et al.Endothelial Progenitor Cells Homing and Renal Repair in Experimental Renovascular Disease, Stem Cells. (2010) 28, no. 6, 1039–1047, 10.1002/stem.426. PMC2958683 20506499

[24] Chade A. R. , Zhu X. , and Lavi R. , et al.Endothelial Progenitor Cells Restore Renal Function in Chronic Experimental Renovascular Disease, Circulation. (2009) 119, no. 4, 547–557, 10.1161/CIRCULATIONAHA.108.788653. PMC2758066 19153272

[25] Zhu X.-Y. , Caceres V. H. U. , Favreau F. D. , Krier J. D. , Lerman A. , and Lerman L. O. , Enhanced Endothelial Progenitor Cell Angiogenic Potency, Present in Early Experimental Renovascular Hypertension, Deteriorates With Disease Duration, Journal of Hypertension. (2011) 29, no. 10, 1972–1979, 10.1097/HJH.0b013e32834ae611. PMC3634099 21873884

[26] Eirin A. , Zhu X. X. , and Li Z. , et al.Endothelial Outgrowth Cells Shift Macrophage Phenotype and Improve Kidney Viability in Swine Renal Artery Stenosis, Arteriosclerosis, Thrombosis, and Vascular Biology. (2013) 33, no. 5, 1006–1013. 10.1161/ATVBAHA.113.301164 PMC3644980 23430615

[27] Chen Z. , Herrmann S. M. S. , and Zhu X. , et al.Preserved Function of Late-Outgrowth Endothelial Cells in Medically Treated Hypertensive Patients Under Well-Controlled Conditions, Hypertension. (2014) 64, no. 4, 808–814, 10.1161/HYPERTENSIONAHA.114.03720. PMC4162760 25047576

[28] Eirin A. , Zhu X. Y , and Krier J. D , et al.Adipose tissue-derived mesenchymal stem cells improve revascularizationoutcomes to restore renal function in swine atherosclerotic renal artery stenosis, Stem Cells. (2012) 30, no. 5, 1030–1041. 10.1002/stem.1047 PMC3694782 22290832

[29] Eirin A. , Siddiqi S. , and Hughes A. G. , et al.Renovascular Disease and Mitochondrial Dysfunction in Human Mesenchymal Stem Cells, Journal of the American Society of Nephrology. (2024) 35, no. 11, 1507–1519, 10.1681/ASN.0000000000000440. PMC11543019 39012704

[30] Eirin A. , Thaler R. , and Glasstetter L. M. , et al.Obesity-Driven Mitochondrial Dysfunction in Human Adipose Tissue-Derived Mesenchymal Stem/Stromal Cells Involves Epigenetic Changes, Cell Death & Disease. (2024) 15, no. 6, 10.1038/s41419-024-06774-8, 387. PMC11144257 38824145

[31] Huang W. , Hong S. , and Zhu X. , et al.Obesity Blunts the Effect of Mesenchymal Stem Cell-Derived Extracellular Vesicles, Kidney International Reports. (2023) 8, no. 9, 1841–1851, 10.1016/j.ekir.2023.06.009. PMC10496020 37705914

[32] Chen X.-J. , Kim S. R. , and Jiang K. , et al.Renovascular Disease Induces Senescence in Renal Scattered Tubular-Like Cells and Impairs Their Reparative Potency, Hypertension. (2021) 77, no. 2, 507–518, 10.1161/HYPERTENSIONAHA.120.16218. PMC7808550 33390051

[33] Nargesi A. A. , Zhu X.-Y. , and Conley S. M. , et al.Renovascular Disease Induces Mitochondrial Damage in Swine Scattered Tubular Cells, American Journal of Physiology-Renal Physiology. (2019) 317, no. 5, F1142–F1153, 10.1152/ajprenal.00276.2019. PMC6879943 31461348

[34] Nargesi A. A. , Zhang L. , and Tang H. , et al.Coexisting Renal Artery Stenosis and Metabolic Syndrome Magnifies Mitochondrial Damage, Aggravating Poststenotic Kidney Injury in Pigs, Journal of Hypertension. (2019) 37, no. 10, 2061–2073, 10.1097/HJH.0000000000002129. PMC6771269 31465309

[35] Farahani R. A. , Zhu X. , Tang H. , Jordan K. L , Lerman L. O , and Eirin A. , Renal Ischemia Alters Expression of Mitochondria-Related Genes and Impairs Mitochondrial Structure and Function in Swine Scattered Tubular-Like Cells, Am J Physiol Renal Physiol. (2020) 319, no. 1, F19–F28. 10.1152/ajprenal.00120.2020 PMC7468829 32463728

[36] Kazeminia S. , Zhu X. Y. , and Tang H. , et al.Renal Ischemia Alters the Transcriptomic and Epigenetic Profile of Inflammatory Genes in Swine Scattered Tubular-Like Cells, Clinical Science. (2023) 137, no. 16, 1265–1283, 10.1042/CS20230555. PMC10644845 37606084

[37] Kazeminia S. , Rajagopalan K. S. , and Zhu X.-Y. , et al.Renal Ischemia Alters the mRNA and miRNA Profile of Vasculature-Related Genes in Scattered Tubular-Like Cells From Female Pigs, American Journal of Physiology-Renal Physiology. (2025) 328, no. 5, F724–F735, 10.1152/ajprenal.00334.2024. PMC12120692 40241609

[38] Kazeminia S. , Kaur B. , and Shivam K. , et al.Renal Ischemia Induces Endoplasmic Reticulum Stress and Impairs the Reparative Potency of Scattered Tubular-Like Cells, American Journal of Nephrology. (2025) 56, no. 5, 657–673, 10.1159/000545795. PMC12353061 40209698

[39] Asahara T. , Murohara T. , and Sullivan A. , et al.Isolation of Putative Progenitor Endothelial Cells for Angiogenesis, Science. (1997) 275, no. 5302, 964–967, 10.1126/science.275.5302.964. 9020076

[40] Khakoo A. Y. and Finkel T. , Endothelial Progenitor Cells, Annual Review of Medicine. (2005) 56, no. 1, 79–101, 10.1146/annurev.med.56.090203.104149. 15660503

[41] Sieveking D. P. , Buckle A. , Celermajer D. S. , and Ng M. K. C. , Strikingly Different Angiogenic Properties of Endothelial Progenitor Cell Subpopulations, Journal of the American College of Cardiology. (2008) 51, no. 6, 660–668, 10.1016/j.jacc.2007.09.059. 18261686

[42] Tagawa S. , Nakanishi C. , and Mori M. , et al.Determination of Early and Late Endothelial Progenitor Cells in Peripheral Circulation and Their Clinical Association With Coronary Artery Disease, International Journal of Vascular Medicine. (2015) 2015, 10.1155/2015/674213, 674213. PMC4588339 26451256

[43] Tura O. , Skinner E. M. , and Barclay G. R. , et al.Late Outgrowth Endothelial Cells Resemble Mature Endothelial Cells and Are Not Derived From Bone Marrow, Stem Cells. (2013) 31, no. 2, 338–348, 10.1002/stem.1280. 23165527

[44] Patschan D. , Krupincza K. , Patschan S. , Zhang Z. , Hamby C. , and Goligorsky M. S. , Dynamics of Mobilization and Homing of Endothelial Progenitor Cells After Acute Renal Ischemia: Modulation by Ischemic Preconditioning, American Journal of Physiology-Renal Physiology. (2006) 291, no. 1, F176–F185, 10.1152/ajprenal.00454.2005. 16478972

[45] Salguero G. , Akin E. , and Templin C. , et al.Renovascular Hypertension by Two-Kidney One-Clip Enhances Endothelial Progenitor Cell Mobilization in a p47Phox-Dependent Manner, Journal of Hypertension. (2008) 26, no. 2, 257–268, 10.1097/HJH.0b013e3282f09f79. 18192840

[46] Tögel F. , Isaac J. , Hu Z. , Weiss K. , and Westenfelder C. , Renal SDF-1 Signals Mobilization and Homing of CXCR4-Positive Cells to the Kidney After Ischemic Injury, Kidney International. (2005) 67, no. 5, 1772–1784, 10.1111/j.1523-1755.2005.00275.x. 15840024

[47] Chade A. R. and Kelsen S. , Reversal of Renal Dysfunction by Targeted Administration of VEGF Into the Stenotic Kidney: A Novel Potential Therapeutic Approach, American Journal of Physiology-Renal Physiology. (2012) 302, no. 10, F1342–F1350, 10.1152/ajprenal.00674.2011. PMC3362061 22357917

[48] Kon K. , et al.Nitric Oxide Synthase Inhibition by N(G)-Nitro-L-Arginine Methyl Ester Retards Vascular Sprouting in Angiogenesis, Microvascular Research. (2003) 65, no. 1, 2–8, 10.1016/S0026-2862(02)00011-0. 12535865

[49] Gupta S. , Verfaillie C. , and Chmielewski D. , et al.Isolation and Characterization of Kidney-Derived Stem Cells, Journal of the American Society of Nephrology. (2006) 17, no. 11, 3028–3040, 10.1681/ASN.2006030275. 16988061

[50] Zhao Q. , Ishibashi M. , Hiasa K.-I. , Tan C. , Takeshita A. , and Egashira K. , Essential Role of Vascular Endothelial Growth Factor in Angiotensin II-Induced Vascular Inflammation and Remodeling, Hypertension. (2004) 44, no. 3, 264–270, 10.1161/01.HYP.0000138688.78906.6b. 15262905

[51] Imanishi T. , Hano T. , and Nishio I. , Angiotensin II Potentiates Vascular Endothelial Growth Factor-Induced Proliferation and Network Formation of Endothelial Progenitor Cells, Hypertension Research. (2004) 27, no. 2, 101–108, 10.1291/hypres.27.101. 15005273

[52] Tamarat R. , Silvestre J.-S.ébastien , Durie M. , and Levy B. I. , Angiotensin II Angiogenic Effect In Vivo Involves Vascular Endothelial Growth Factor- and Inflammation-Related Pathways, Laboratory Investigation. (2002) 82, no. 6, 747–756, 10.1097/01.LAB.0000017372.76297.EB. 12065685

[53] Sugiura T. , Kondo T. , and Kureishi-Bando Y. , et al.Nifedipine Improves Endothelial Function: Role of Endothelial Progenitor Cells, Hypertension. (2008) 52, no. 3, 491–498, 10.1161/HYPERTENSIONAHA.108.111914. 18645050

[54] Dominici M. , Blanc K. L. , and Mueller I. , et al.Minimal Criteria for Defining Multipotent Mesenchymal Stromal Cells. The International Society for Cellular Therapy Position Statement, Cytotherapy. (2006) 8, no. 4, 315–317, 10.1080/14653240600855905. 16923606

[55] Kramann R. and Humphreys B. D. , Kidney Pericytes: Roles in Regeneration and Fibrosis, Seminars in Nephrology. (2014) 34, no. 4, 374–383, 10.1016/j.semnephrol.2014.06.004. PMC4163198 25217266

[56] Asanuma H. , Meldrum D. R. , and Meldrum K. K. , Therapeutic Applications of Mesenchymal Stem Cells to Repair Kidney Injury, Journal of Urology. (2010) 184, no. 1, 26–33, 10.1016/j.juro.2010.03.050. 20478602

[57] Kramann R. , Wongboonsin J. , Chang-Panesso M. , Machado F. G. , and Humphreys B. D. , Gli1+ Pericyte Loss Induces Capillary Rarefaction and Proximal Tubular Injury, Journal of the American Society of Nephrology. (2017) 28, no. 3, 776–784, 10.1681/ASN.2016030297. PMC5328159 27624490

[58] Sengun E. , Wolfs T. G. A. M. , and van Bruggen V. L. E. , et al.Umbilical Cord-Mesenchymal Stem Cells Induce a Memory Phenotype in CD4(+) T Cells, Frontiers in Immunology. (2023) 14, 10.3389/fimmu.2023.1128359, 1128359. PMC10318901 37409122

[59] van Koppen A. , Joles J. A. , and van Balkom B. W. M. , et al.Human Embryonic Mesenchymal Stem Cell-Derived Conditioned Medium Rescues Kidney Function in Rats with Established Chronic Kidney Disease, PLoS ONE. (2012) 7, no. 6, 10.1371/journal.pone.0038746. PMC3378606 22723882

[60] Song T. , Zhu X.-Y. , and Eirin A. , et al.Exogenous Pericyte Delivery Protects the Mouse Kidney From Chronic Ischemic Injury, American Journal of Physiology-Renal Physiology. (2022) 323, no. 5, F527–F538, 10.1152/ajprenal.00064.2022. PMC9602803 36049063

[61] Eirin A. , Zhu X.-Y. , and Puranik A. S. , et al.Mesenchymal Stem Cell-Derived Extracellular Vesicles Attenuate Kidney Inflammation, Kidney International. (2017) 92, no. 1, 114–124, 10.1016/j.kint.2016.12.023. PMC5483390 28242034

[62] Eirin A. , Riester S. M. , and Zhu X.-Y. , et al.MicroRNA and mRNA Cargo of Extracellular Vesicles From Porcine Adipose Tissue-Derived Mesenchymal Stem Cells, Gene. (2014) 551, no. 1, 55–64, 10.1016/j.gene.2014.08.041. PMC4174680 25158130

[63] Song T. , Eirin A. , and Zhu X. , et al.Mesenchymal Stem Cell-Derived Extracellular Vesicles Induce Regulatory T Cells to Ameliorate Chronic Kidney Injury, Hypertension. (2020) 75, no. 5, 1223–1232, 10.1161/HYPERTENSIONAHA.119.14546. PMC7219723 32223383

[64] Saad A. , Zhu X.-Y. , and Herrmann S. , et al.Adipose-Derived Mesenchymal Stem Cells From Patients With Atherosclerotic Renovascular Disease Have Increased DNA Damage and Reduced Angiogenesis that Can Be Modified by Hypoxia, Stem Cell Research & Therapy. (2016) 7, no. 1, 10.1186/s13287-016-0389-x, 128. PMC5016873 27612459

[65] Smeets B. , Boor P. , and Dijkman H. , et al.Proximal Tubular Cells Contain a Phenotypically Distinct, Scattered Cell Population Involved in Tubular Regeneration, The Journal of Pathology. (2013) 229, no. 5, 645–659, 10.1002/path.4125. PMC3951144 23124355

[66] Romagnani P. and Remuzzi G. , CD133+ Renal Stem Cells Always Co-Express CD24 in Adult Human Kidney Tissue, Stem Cell Research. (2014) 12, no. 3, 828–829, 10.1016/j.scr.2013.12.011. 24467938

[67] Kumar S. , Liu J. , and Pang P. , et al.Sox9 Activation Highlights a Cellular Pathway of Renal Repair in the Acutely Injured Mammalian Kidney, Cell Reports. (2015) 12, no. 8, 1325–1338, 10.1016/j.celrep.2015.07.034. 26279573

[68] Genheimer C. W. , Ilagan R. M. , and Spencer T. , et al.Molecular Characterization of the Regenerative Response Induced by Intrarenal Transplantation of Selected Renal Cells in a Rodent Model of Chronic Kidney Disease, Cells Tissues Organs. (2012) 196, no. 4, 374–384, 10.1159/000336028. 22584161

[69] Kang H. M. , Huang S. , Reidy K. , Han S. H. , Chinga F. , and Susztak K. , Sox9-Positive Progenitor Cells Play a Key Role in Renal Tubule Epithelial Regeneration in Mice, Cell Reports. (2016) 14, no. 4, 861–871, 10.1016/j.celrep.2015.12.071. PMC4854207 26776520

[70] Sagrinati C. , Netti G. S. , and Mazzinghi B. , et al.Isolation and Characterization of Multipotent Progenitor Cells From the Bowman’s Capsule of Adult Human Kidneys, Journal of the American Society of Nephrology. (2006) 17, no. 9, 2443–2456, 10.1681/ASN.2006010089. 16885410

[71] Hansson J. , Hultenby K. , and Cramnert C. , et al.Evidence for a Morphologically Distinct and Functionally Robust Cell Type in the Proximal Tubules of Human Kidney, Human Pathology. (2014) 45, no. 2, 382–393, 10.1016/j.humpath.2013.10.003. 24439225

[72] Kemper K. , et al.The AC133 Epitope, but not the CD133 Protein, Is Lost upon Cancer Stem Cell Differentiation, Cancer Research. (2010) 70, no. 2, 719–729, 10.1158/0008-5472.CAN-09-1820. 20068153

[73] Shirasawa T. , Akashi T. , Sakamoto K. , Takahashi H. , Maruyama N. , and Hirokawa K. , Gene Expression of CD24 Core Peptide Molecule in Developing Brain and Developing Non-Neural Tissues, Developmental Dynamics. (1993) 198, no. 1, 1–13, 10.1002/aja.1001980102. 8292828

[74] Kramann R. , Kusaba T. , and Humphreys B. D. , Who Regenerates the Kidney Tubule?, Nephrology Dialysis and Transplant. (2015) 30, no. 6, 903–910. 10.1093/ndt/gfu281 PMC4438740 25155054

[75] Berger K. , Jörg-M. Bangen L. Hammerich , Liedtke C. , J.C. , rgen Floege B. Smeets , and Moeller M. J. , Origin of Regenerating Tubular Cells After Acute Kidney Injury, Proceedings of the National Academy of Sciences. (2014) 111, no. 4, 1533–1538, 10.1073/pnas.1316177111. PMC3910599 24474779

[76] Romagnani P. , Lasagni L. , and Remuzzi G. , Renal Progenitors: An Evolutionary Conserved Strategy for Kidney Regeneration, Nature Reviews Nephrology. (2013) 9, no. 3, 137–146, 10.1038/nrneph.2012.290. 23338209

[77] Angelotti M. L. , Ronconi E. , and Ballerini L. , et al.Characterization of Renal Progenitors Committed Toward Tubular Lineage and Their Regenerative Potential in Renal Tubular Injury, Stem Cells. (2012) 30, no. 8, 1714–1725, 10.1002/stem.1130. 22628275

[78] Lazzeri E. , Angelotti M. L. , and Peired A. , et al.Endocycle-Related Tubular Cell Hypertrophy and Progenitor Proliferation Recover Renal Function After Acute Kidney Injury, Nature Communications. (2018) 9, no. 1, 10.1038/s41467-018-03753-4, 1344. PMC5890293 29632300

[79] Bussolati B. , Bruno S. , and Grange C. , et al.Isolation of Renal Progenitor Cells from Adult Human Kidney, The American Journal of Pathology. (2005) 166, no. 2, 545–555, 10.1016/S0002-9440(10)62276-6. PMC1602314 15681837

[80] Grange C. , Moggio A. , Tapparo M. , Porta S. , Camussi G. , and Bussolati B. , Protective Effect and Localization by Optical Imaging of Human Renal CD133+ Progenitor Cells in an Acute Kidney Injury Model, Physiological Reports. (2014) 2, no. 5, 10.14814/phy2.12009. PMC4098737 24793983

[81] Lazzeri E. , Crescioli C. , and Ronconi E. , et al.Regenerative Potential of Embryonic Renal Multipotent Progenitors in Acute Renal Failure, Journal of the American Society of Nephrology. (2007) 18, no. 12, 3128–3138, 10.1681/ASN.2007020210. 17978305

[82] Aggarwal S. , Grange C. , Iampietro C. , Camussi G. , and Bussolati B. , Human CD133+ Renal Progenitor Cells Induce Erythropoietin Production and Limit Fibrosis After Acute Tubular Injury, Scientific Reports. (2016) 6, no. 1, 10.1038/srep37270, 37270. PMC5112528 27853265

[83] Bussolati B. , Lauritano C. , Moggio A. , Collino F. , Mazzone M. , and Camussi G. , Renal CD133(+)/CD73(+) Progenitors Produce Erythropoietin Under Hypoxia and Prolyl Hydroxylase Inhibition, Journal of the American Society of Nephrology. (2013) 24, no. 8, 1234–1241, 10.1681/ASN.2012080772. PMC3736703 23661806

[84] Sallustio F. , Curci C. , and Aloisi A. , et al.Inhibin-A and Decorin Secreted by Human Adult Renal Stem/Progenitor Cells Through the TLR2 Engagement Induce Renal Tubular Cell Regeneration, Scientific Reports. (2017) 7, no. 1, 10.1038/s41598-017-08474-0, 8225. PMC5557965 28811645

[85] Wang B. , Han J. , Elisseeff J. H. , and Demaria M. , The Senescence-Associated Secretory Phenotype and Its Physiological and Pathological Implications, Nature Reviews Molecular Cell Biology. (2024) 25, no. 12, 958–978, 10.1038/s41580-024-00727-x. 38654098

[86] Solaini G. , Baracca A. , Lenaz G. , and Sgarbi G. , Hypoxia and Mitochondrial Oxidative Metabolism, Biochimica et Biophysica Acta (BBA) - Bioenergetics. (2010) 1797, no. 6-7, 1171–1177, 10.1016/j.bbabio.2010.02.011. 20153717

[87] Grote K. , Flach I. , and Luchtefeld M. , et al.Mechanical Stretch Enhances mRNA Expression and Proenzyme Release of Matrix Metalloproteinase-2 (MMP-2) via NAD(P)H Oxidase-Derived Reactive Oxygen Species, Circulation Research. (2003) 92, no. 11, e80–e86, 10.1161/01.RES.0000077044.60138.7C. 12750313

[88] Touyz R. M. , Yao G. , and Schiffrin E. L. , et al.c-Src Induces Phosphorylation and Translocation of p47Phox: Role in Superoxide Generation by Angiotensin II in Human Vascular Smooth Muscle Cells, Arteriosclerosis, Thrombosis, and Vascular Biology. (2003) 23, no. 6, 981–987. 10.1161/01.ATV.0000069236.27911.68 12663375

[89] Piotrowska A. , Chmielewska M. , Andrzejewski W. , Dziegiel P. , and Podhorska-Okolow M. , Influence of Angiotensin II on Cell Viability and Apoptosis in Rat Renal Proximal Tubular Epithelial Cells in In Vitro Studies, Journal of the Renin-Angiotensin-Aldosterone System. (2020) 21, no. 3, 10.1177/1470320320949850, 1470320320949850. PMC7649907 32962526

[90] Shadel G. S. and Horvath T. L. , Mitochondrial ROS Signaling in Organismal Homeostasis, Cell. (2015) 163, no. 3, 560–569, 10.1016/j.cell.2015.10.001. PMC4634671 26496603

[91] Nunnari J. and Suomalainen A. , Mitochondria: In Sickness and in Health, Cell. (2012) 148, no. 6, 1145–1159, 10.1016/j.cell.2012.02.035. PMC5381524 22424226

[92] Hetz C. , Zhang K. , and Kaufman R. J. , Mechanisms, Regulation and Functions of the Unfolded Protein Response, Nature Reviews Molecular Cell Biology. (2020) 21, no. 8, 421–438, 10.1038/s41580-020-0250-z. PMC8867924 32457508

[93] Zhang D. , Lu H. , and Chen Z. , et al.High Glucose Induces the Aging of Mesenchymal Stem Cells via Akt/mTOR Signaling, Molecular Medicine Reports. (2017) 16, no. 2, 1685–1690, 10.3892/mmr.2017.6832. PMC5562095 28656269

[94] López-Otín C. , Blasco M. A. , Partridge L. , Serrano M. , and Kroemer G. , The Hallmarks of Aging, Cell. (2013) 153, no. 6, 1194–1217, 10.1016/j.cell.2013.05.039. PMC3836174 23746838

[95] Heida N.-M. , Müller J.-P. , and Cheng I.-F. , et al.Effects of Obesity and Weight Loss on the Functional Properties of Early Outgrowth Endothelial Progenitor Cells, Journal of the American College of Cardiology. (2010) 55, no. 4, 357–367, 10.1016/j.jacc.2009.09.031. 20117442

[96] Tepper O. M. , Galiano R. D. , and Capla J. M. , et al.Human Endothelial Progenitor Cells From Type II Diabetics Exhibit Impaired Proliferation, Adhesion, and Incorporation Into Vascular Structures, Circulation. (2002) 106, no. 22, 2781–2786, 10.1161/01.CIR.0000039526.42991.93. 12451003

[97] Wahba I. M. and Mak R. H. , Obesity and Obesity-Initiated Metabolic Syndrome, Clinical Journal of the American Society of Nephrology. (2007) 2, no. 3, 550–562, 10.2215/CJN.04071206. 17699463

[98] Hsu C.-Y. , McCulloch C. E. , Iribarren C. , Darbinian J. , and Go A. S. , Body Mass Index and Risk for End-Stage Renal Disease, Annals of Internal Medicine. (2006) 144, no. 1, 21–28, 10.7326/0003-4819-144-1-200601030-00006. 16389251

[99] Morton J. I. , Liew D. , McDonald S. P. , Shaw J. E. , and Magliano D. J. , The Association Between Age of Onset of Type 2 Diabetes and the Long-Term Risk of End-Stage Kidney Disease: A National Registry Study, Diabetes Care. (2020) 43, no. 8, 1788–1795, 10.2337/dc20-0352. 32540924

[100] Thomas M. C. , Cooper M. E. , and Zimmet P. , Changing Epidemiology of Type 2 Diabetes Mellitus and Associated Chronic Kidney Disease, Nature Reviews Nephrology. (2016) 12, no. 2, 73–81, 10.1038/nrneph.2015.173. 26553517

[101] Munichoodappa C. , D’Elia J. A. , Libertino J. A. , Gleason R. E. , and Christlieb A. R. , Renal Artery Stenosis in Hypertensive Diabetics, Journal of Urology. (1979) 121, no. 5, 555–558, 10.1016/S0022-5347(17)56875-5. 439246

[102] Ritchie C. M. , Mcllrath E. , Hadden D. R. , Weaver J. A. , Kennedy L. , and Atkinson A. B. , Renal Artery Stenosis in Hypertensive Diabetic Patients, Diabetic Medicine. (1988) 5, no. 3, 265–267, 10.1111/j.1464-5491.1988.tb00982.x. 2967149

[103] Nicholls A. J. , The Impact of Atherosclerotic Renovascular Disease on Diabetic Renal Failure, Diabetic Medicine. (2002) 19, no. 11, 889–894, 10.1046/j.1464-5491.2002.00813.x. 12421424

[104] Lin L. , Tan W. , Pan X. , Tian E. , Wu Z. , and Yang J. , Metabolic Syndrome-Related Kidney Injury: A Review and Update, Frontiers in Endocrinology. (2022) 13, 10.3389/fendo.2022.904001, 904001. PMC9261267 35813613

[105] Postma C. T. , Klappe E. M. , Dekker H. M. , and Thien T. , The Prevalence of Renal Artery Stenosis Among Patients With Diabetes Mellitus, European Journal of Internal Medicine. (2012) 23, no. 7, 639–642, 10.1016/j.ejim.2012.06.003. 22939809

[106] Song T. , Zhao Y. , and Zhu X. , et al.Superimposition of Metabolic Syndrome Magnifies Post-Stenotic Kidney Injury in Dyslipidemic Pigs, American Journal of Translational Research. (2021) 13, no. 8, 8965–8976. PMC8430139 34540008

[107] Tsai T.-H. , Chai H.-T. , and Sun C.-K. , et al.Obesity Suppresses Circulating Level and Function of Endothelial Progenitor Cells and Heart Function, Journal of Translational Medicine. (2012) 10, no. 1, 10.1186/1479-5876-10-137, 137. PMC3543390 22747715

[108] Noor R. , Shuaib U. , and Wang C. X. , et al.High-Density Lipoprotein Cholesterol Regulates Endothelial Progenitor Cells by Increasing eNOS and Preventing Apoptosis, Atherosclerosis. (2007) 192, no. 1, 92–99, 10.1016/j.atherosclerosis.2006.06.023. 16884727

[109] Hörtenhuber T. , Rami-Mehar B. , and Satler M. , et al.Endothelial Progenitor Cells Are Related to Glycemic Control in Children With Type 1 Diabetes Over Time, Diabetes Care. (2013) 36, no. 6, 1647–1653, 10.2337/dc12-1206. PMC3661838 23340890

[110] Xing L. , Mondesir R. , and Glasstetter L. M. , et al.The Impact of Obesity on Autophagy in Human Adipose-Derived Mesenchymal Stromal Cells, Cell Transplantation. (2025) 34, 10.1177/09636897251323339, 9636897251323339. PMC11930488 40116436

[111] Pawar A. S. , Eirin A. , and Krier J. D. , et al.Alterations in Genetic and Protein Content of Swine Adipose Tissue-Derived Mesenchymal Stem Cells in the Metabolic Syndrome, Stem Cell Research. (2019) 37, 10.1016/j.scr.2019.101423, 101423. PMC6545577 30933719

[112] Pawar A. S. , Eirin A. , Tang H. , Zhu X.-Y. , Lerman A. , and Lerman L. O. , Upregulated Tumor Necrosis Factor-Alpha Transcriptome and Proteome in Adipose Tissue-Derived Mesenchymal Stem Cells From Pigs With Metabolic Syndrome, Cytokine. (2020) 130, 10.1016/j.cyto.2020.155080, 155080. PMC7529712 32240922

[113] Conley S. M. , Zhu X.-Y. , and Eirin A. , et al.Metabolic Syndrome Alters Expression of Insulin Signaling-Related Genes in Swine Mesenchymal Stem Cells, Gene. (2018) 644, 101–106, 10.1016/j.gene.2017.10.086. PMC5771951 29101070

[114] Conley S. M. , Shook J. E. , and Zhu X.-Y. , et al.Metabolic Syndrome Induces Release of Smaller Extracellular Vesicles From Porcine Mesenchymal Stem Cells, Cell Transplantation. (2019) 28, no. 9-10, 1271–1278, 10.1177/0963689719860840. PMC6767891 31250656

[115] Kushner E. J. , Van Guilder G. P. , MacEneaney O. J. , Cech J. N. , Stauffer B. L. , and DeSouza C. A. , Aging and Endothelial Progenitor Cell Telomere Length in Healthy Men, Clinical Chemistry and Laboratory Medicine. (2009) 47, no. 1, 47–50, 10.1515/CCLM.2009.016. PMC2646422 19055473

[116] Kaur I. , Rawal P. , and Rohilla S. , et al.Endothelial Progenitor Cells From Aged Subjects Display Decreased Expression of Sirtuin 1, Angiogenic Functions, and Increased Senescence, Cell Biology International. (2018) 42, no. 9, 1212–1220, 10.1002/cbin.10999. 29851177

[117] Reskiawan R. , Kadir A. , and Alwjwaj M. , et al.Inhibition of Oxidative Stress Delays Senescence and Augments Functional Capacity of Endothelial Progenitor Cells, Brain Research. (2022) 1787, 10.1016/j.brainres.2022.147925, 147925. 35469846

[118] Aicher A. , Heeschen C. , and Mildner-Rihm C. , et al.Essential Role of Endothelial Nitric Oxide Synthase for Mobilization of Stem and Progenitor Cells, Nature Medicine. (2003) 9, no. 11, 1370–1376, 10.1038/nm948. 14556003

[119] Weng Z. , Wang Y. , and Ouchi T. , et al.Mesenchymal Stem/Stromal Cell Senescence: Hallmarks, Mechanisms, and Combating Strategies, Stem Cells Translational Medicine. (2022) 11, no. 4, 356–371, 10.1093/stcltm/szac004. PMC9052415 35485439

[120] Strehlow K. , Werner N. , and Berweiler J. , et al.Estrogen Increases Bone Marrow-Derived Endothelial Progenitor Cell Production and Diminishes Neointima Formation, Circulation. (2003) 107, no. 24, 3059–3065, 10.1161/01.CIR.0000077911.81151.30. 12810616

[121] Imanishi T. , Hano T. , and Nishio I. , Estrogen Reduces Endothelial Progenitor Cell Senescence Through Augmentation of Telomerase Activity, Journal of Hypertension. (2005) 23, no. 9, 1699–1706, 10.1097/01.hjh.0000176788.12376.20. 16093915

[122] Masuda H. , Kalka C. , and Takahashi T. , et al.Estrogen-Mediated Endothelial Progenitor Cell Biology and Kinetics For Physiological Postnatal Vasculogenesis, Circulation Research. (2007) 101, no. 6, 598–606, 10.1161/CIRCRESAHA.106.144006. 17656679

[123] Rousseau A. , Ayoubi F. , and Deveaux C. , et al.Impact of Age and Gender Interaction on Circulating Endothelial Progenitor Cells in Healthy Subjects, Fertility and Sterility. (2010) 93, no. 3, 843–846, 10.1016/j.fertnstert.2008.10.062. 19111297

[124] Raposo G. and Stoorvogel W. , Extracellular Vesicles: Exosomes, Microvesicles, and Friends, Journal of Cell Biology. (2013) 200, no. 4, 373–383, 10.1083/jcb.201211138. PMC3575529 23420871

[125] van Niel G. , D’Angelo G. , and Raposo G. , Shedding Light on the Cell Biology of Extracellular Vesicles, Nature Reviews Molecular Cell Biology. (2018) 19, no. 4, 213–228, 10.1038/nrm.2017.125. 29339798

[126] Théry C. , Witwer K. W. , and Aikawa E. , et al.Minimal Information for Studies of Extracellular Vesicles 2018 (MISEV2018): A Position Statement of the International Society for Extracellular Vesicles and Update of the MISEV2014 Guidelines, Journal of Extracellular Vesicles. (2018) 7, no. 1, 10.1080/20013078.2018.1535750, 1535750. PMC6322352 30637094

[127] Valadi H. , Ekström K. , Bossios A. , Sjöstrand M. , Lee J. J. , and Lötvall J. O. , Exosome-Mediated Transfer of mRNAs and microRNAs Is a Novel Mechanism of Genetic Exchange Between Cells, Nature Cell Biology. (2007) 9, no. 6, 654–659, 10.1038/ncb1596. 17486113

[128] Yáñez-Mó M. , Siljander P. R.-M. , and Andreu Z. , et al.Biological Properties of Extracellular Vesicles and Their Physiological Functions, Journal of Extracellular Vesicles. (2015) 4, no. 1, 10.3402/jev.v4.27066, 27066. PMC4433489 25979354

[129] Mulcahy L. A. , Pink R. C. , and Carter D. R. F. , Routes and Mechanisms of Extracellular Vesicle Uptake, Journal of Extracellular Vesicles. (2014) 3, no. 1, 10.3402/jev.v3.24641, 3. PMC4122821 25143819

[130] Mathieu M. , Martin-Jaular L. , Lavieu G. , and Théry C. , Specificities of Secretion and Uptake of Exosomes and Other Extracellular Vesicles for Cell-to-Cell Communication, Nature Cell Biology. (2019) 21, no. 1, 9–17, 10.1038/s41556-018-0250-9. 30602770

[131] Szeto H. H. , First-in-Class Cardiolipin-Protective Compound as a Therapeutic Agent to Restore Mitochondrial Bioenergetics, British Journal of Pharmacology. (2014) 171, no. 8, 2029–2050, 10.1111/bph.12461. PMC3976620 24117165

[132] Farooqui N. , Mohan A. , and Isik B. , et al.Effect of Hypoxia Preconditioning on the Regenerative Capacity of Adipose Tissue Derived Mesenchymal Stem Cells in a Model of Renal Artery Stenosis, Stem Cells. (2023) 41, no. 1, 50–63, 10.1093/stmcls/sxac073. PMC9887092 36250949

[133] Franca C. N. , Amaral J. B. , and Tuleta I. D. , et al.Challenges Facing the Use of Endothelial Progenitor Cells in Stem Cell Therapies, Critical Reviews in Eukaryotic Gene Expression. (2016) 26, no. 2, 161–162, 10.1615/CritRevEukaryotGeneExpr.2016016325. 27480778

[134] Tolar J. , Nauta A. J. , and Osborn M. J. , et al.Sarcoma Derived from Cultured Mesenchymal Stem Cells, Stem Cells. (2007) 25, no. 2, 371–379, 10.1634/stemcells.2005-0620. 17038675

[135] Thirabanjasak D. , Tantiwongse K. , and Thorner P. S. , Angiomyeloproliferative Lesions Following Autologous Stem Cell Therapy, Journal of the American Society of Nephrology. (2010) 21, no. 7, 1218–1222, 10.1681/ASN.2009111156. PMC3152236 20558536

[136] Takahashi K. and Yamanaka S. , Induction of Pluripotent Stem Cells From Mouse Embryonic and Adult Fibroblast Cultures by Defined Factors, Cell. (2006) 126, no. 4, 663–676, 10.1016/j.cell.2006.07.024. 16904174

[137] Takahashi K. , Tanabe K. , and Ohnuki M. , et al.Induction of Pluripotent Stem Cells From Adult Human Fibroblasts by Defined Factors, Cell. (2007) 131, no. 5, 861–872, 10.1016/j.cell.2007.11.019. 18035408

[138] Takasato M. , Er P. X. , and Chiu H. S. , et al.Kidney Organoids From Human iPS Cells Contain Multiple Lineages and Model Human Nephrogenesis, Nature. (2015) 526, no. 7574, 564–568, 10.1038/nature15695. 26444236

[139] Morizane R. , Lam A. Q. , Freedman B. S. , Kishi S. , Valerius M. T. , and Bonventre J. V. , Nephron Organoids Derived From Human Pluripotent Stem Cells Model Kidney Development and Injury, Nature Biotechnology. (2015) 33, no. 11, 1193–1200, 10.1038/nbt.3392. PMC4747858 26458176

[140] Frobel J. , Hemeda H. , and Lenz M. , et al.Epigenetic Rejuvenation of Mesenchymal Stromal Cells Derived From Induced Pluripotent Stem Cells, Stem Cell Reports. (2014) 3, no. 3, 414–422, 10.1016/j.stemcr.2014.07.003. PMC4266008 25241740

[141] Riazifar M. , Pone E. J. , Lötvall J. , and Zhao W. , Stem Cell Extracellular Vesicles: Extended Messages of Regeneration, Annual Review of Pharmacology and Toxicology. (2017) 57, no. 1, 125–154, 10.1146/annurev-pharmtox-061616-030146. PMC5360275 27814025

[142] Munk A. , Duvald C. Søndergaard , and Pedersen M. , et al.Dosing Limitation for Intra-Renal Arterial Infusion of Mesenchymal Stromal Cells, International Journal of Molecular Sciences. (2022) 23, no. 15, 10.3390/ijms23158268, 8268. PMC9368110 35955404

[143] Robb K. , Fitzgerald J. , Barry F. , and Viswanathan S. , Mesenchymal Stromal Cell Therapy: Progress in Manufacturing and Assessments of Potency, Cytotherapy. (2019) 21, no. 3, 289–306, 10.1016/j.jcyt.2018.10.014. 30528726

[144] Galipeau J. and Sensebe L. , Mesenchymal Stromal Cells: Clinical Challenges and Therapeutic Opportunities, Cell Stem Cell. (2018) 22, no. 6, 824–833, 10.1016/j.stem.2018.05.004. PMC6434696 29859173

[145] Mendicino M. , Bailey A. M. , Wonnacott K. , Puri R. K. , and Bauer S. R. , MSC-Based Product Characterization for Clinical Trials: An FDA Perspective, Cell Stem Cell. (2014) 14, no. 2, 141–145, 10.1016/j.stem.2014.01.013. 24506881

